# Piezo1–Pannexin1 complex couples force detection to ATP secretion in cholangiocytes

**DOI:** 10.1085/jgp.202112871

**Published:** 2021-10-25

**Authors:** Angélique Desplat, Virginie Penalba, Emeline Gros, Thibaud Parpaite, Bertrand Coste, Patrick Delmas

**Affiliations:** Aix-Marseille-Université, Centre National de la Recherche Scientifique, Laboratoire de Neurosciences Cognitives, UMR 7291, CS80011, Marseille, France

## Abstract

Cholangiocytes actively contribute to the final composition of secreted bile. These cells are exposed to abnormal mechanical stimuli during obstructive cholestasis, which has a deep impact on their function. However, the effects of mechanical insults on cholangiocyte function are not understood. Combining gene silencing and pharmacological assays with live calcium imaging, we probed molecular candidates essential for coupling mechanical force to ATP secretion in mouse cholangiocytes. We show that Piezo1 and Pannexin1 are necessary for eliciting the downstream effects of mechanical stress. By mediating a rise in intracellular Ca^2+^, Piezo1 acts as a mechanosensor responsible for translating cell swelling into activation of Panx1, which triggers ATP release and subsequent signal amplification through P2X4R. Co-immunoprecipitation and pull-down assays indicated physical interaction between Piezo1 and Panx1, which leads to stable plasma membrane complexes. Piezo1–Panx1–P2X4R ATP release pathway could be reconstituted in HEK Piezo1 KO cells. Thus, our data suggest that Piezo1 and Panx1 can form a functional signaling complex that controls force-induced ATP secretion in cholangiocytes. These findings may foster the development of novel therapeutic strategies for biliary diseases.

## Teaser

Desplat et al. show that the Piezo1 channel mediates Ca^2+^ entry into mouse cholangiocytes as a result of mechanical stress due to cell swelling. Piezo1 thus activates and forms complexes with Pannexin1, which result in force-induced ATP release and signal amplification through P2X4R.

## Introduction

Cholangiocytes are epithelial cells that line the intra-hepatic ducts of the biliary tree and serve important functions in bile formation. These cells modify the composition of primary hepatocyte-derived bile through a sequence of secretory and reabsorptive processes, critical for digestion and absorption of aliments (Banales et al., 2019). Although they account for a minor subset of the total hepatic cell population, they generate up to ≈40% of the bile volume formation (Boyer, 2013).

Cholangiocyte functions are regulated by multiple chemical factors, including hormones, peptides, nucleotides, and bile acids, through a variety of intracellular signaling pathways, ion channels, transporters, and receptors (Tabibian et al., 2013). Besides being targets for chemical mediators, cholangiocytes are also exposed to mechanical forces, including osmotic pressure and shear stress due to changes in bile composition and flow. Pathological conditions, associated with biliary obstruction, are also responsible for abnormal mechanical tension applied onto the cholangiocyte plasma membrane. Primary sclerosing cholangitis is an exemplar case, where fibro-obliteration of the bile ducts is responsible for liver cirrhosis (Pinzani and Luong, 2018). Biliary outflow obstruction leads to increased biliary pressure, triggering pressure-induced maladaptive signaling in cholangiocytes that strengthens the disease progression (Maroni et al., 2015).

Mechanosensitive signaling has therefore emerged as a regulatory mechanism for the functions of cholangiocytes in both health and disease. However, the initiating steps translating mechanical forces into biochemical messages are not quite understood. Previous in vitro studies have demonstrated that mechanical strain and shear stress induce an elevation of intracellular Ca^2+^ concentration ([Ca^2+^]_i_) through an increase in plasma membrane permeability to Ca^2+^ ions. These responses were proposed to depend on the primary cilium (Masyuk et al., 2008; Larusso and Masyuk, 2011; Mansini et al., 2018). Primary cilium’s ability to integrate mechanical signals relies on the presence of a variety of ciliary-associated proteins that act as mechanotransducers. Among the various known mechanosensitive proteins, the importance of transient receptor potential (TRP) channels has been extensively studied. Changes in bile flow has been proposed to activate a ciliary complex formed by the association of TRPP1 (polycystin-1) and TRPP2 (polycystin-2; Masyuk et al., 2006), two members of the TRPP (polycystin) protein family thought to hold mechanosensory properties (Nauli et al., 2003). On the other hand, the cilia-associated TRPV4 channel, a well-established osmosensor (Liedtke, 2005), has been shown to transduce changes in bile tonicity in cholangiocytes (Gradilone et al., 2007). Activation of these mechanosensitive channels promotes ATP release, which in turn, through autocrine/paracrine stimulation of one or more purinergic receptors, further increases [Ca^2+^]_i_ (Feranchak and Fitz, 2002). Purinergic signals promote transepithelial secretion of anions, water, and HCO_3_^−^, resulting in dilution and alkalinization of bile (Roman et al.,1999; Tabibian et al., 2013).

Therefore, there has emerged from the existing data a model in which mechanotransducers coordinate ductal bile modification through intracellular Ca^2+^ signaling pathways and ATP release. However, neither the mechanotransducer apparatus nor the ATP-release pathway has been definitely identified. Accordingly, the present study aims at dissecting the molecular mechanisms that couple mechanosensitive transducers to ATP secretion in cholangiocytes from mouse liver. We found that cholangiocytes from intrahepatic bile duct units (IBDUs) sense mechanical strain through the activation of Piezo1, a Ca^2+^-permeable cation channel directly gated by mechanical forces (Coste et al., 2010; Ranade et al., 2015; Parpaite and Coste, 2017). The ensuing ATP release requires Pannexin1 (Panx1), a member of the “gap junction” protein family that forms nonjunctional membrane channels permeable to ATP (Dahl, 2015; Taruno, 2018). Both proteins show potential for physical interaction. Thus, Piezo1 and Panx1 constitute a novel pathway to sense and signal mechanical cues in cholangiocytes. These proteins may be important new targets to regulate bile secretion in the treatment of cholestatic liver diseases.

## Materials and methods

### Animals

All animals were used in accordance with the European Community guiding in the care and use of animals (2010/63/UE). Experiments were performed in accordance with the French government regulations.

### Osmotic solutions

The Krebs solution consisted of (in mM) 130 NaCl, 3 KCl, 1 MgCl_2_, 10 HEPES, 10 glucose, and 2.5 CaCl_2_ (290 ± 5 mOsm ⋅ liter^−1^). The isotonic external solution consisted of (in mM) 65 NaCl, 3 KCl, 1 MgCl_2_, 2.5 CaCl_2_, 10 HEPES, 10 glucose, and 120 mannitol (290 ± 5 mOsm ⋅ liter^−1^). The hypotonic solution (160 ± 5 mOsm ⋅ liter^−1^) was generated by removing mannitol to the isotonic external solution, whereas the hypertonic solution (460 ± 5 mOsm ⋅ liter^−1^) was prepared by adding mannitol to the Krebs solution. Ca^2+^-free solutions were prepared by omitting CaCl_2_, and no EGTA was added.

### Primary cultures from mouse IBDUs

Primary cultures of cholangiocytes from IBDUs were obtained mainly as described (Mennone et al., 1995). Male C57BL6 mice aged 10–20 wk were used for isolation of the biliary tree. Mice were anesthetized with isoflurane and sacrificed by decapitation. The liver was perfused through the portal vein for 5 min with PBS and then for 20 min with PBS containing 1 mg/ml collagenase D (Sigma-Aldrich; #COLLD-RO) and 100 µM CaCl_2_. After careful removal of the Glisson’s capsule, the intrahepatic biliary tree was isolated by repeated washes in PBS and thinly minced with scissors in PBS supplemented with 2 mg/ml collagenase D, 0.32 mg/ml pronase (Sigma-Aldrich; #PRON-RO), 0.04 mg/ml deoxyribonuclease (DNase; Sigma-Aldrich; #DN25), 1% FBS (Gibco; #10270), and 1% penicillin/streptomycin (P/S; Gibco; #15140-122). These pieces were digested for 45 min at 37°C and then mechanically dissociated and sifted in a 100-µm cell-strainer. After centrifugation, cells were resuspended in PBS supplemented with 2 mg/ml collagenase D, 0.34 mg/ml hyaluronidase (Sigma-Aldrich; #H3506), 0.04 mg/ml DNase, 1% FBS, and 1% P/S. Cells were incubated for 20 min at 37°C. Finally, cells were seeded on rat-tail type-I collagen–coated Petri dishes (Corning) or coverslips with Dulbecco’s modified Eagle’s medium/F-12 medium (Gibco; #31331-028) supplemented with 3.6% heat-inactivated FBS, 1% P/S, and 1‰ gentamicin (Gibco; #15750-037) and incubated at 37°C in a CO_2_-equilibrated incubator. A small subset of cells, negative for cytokeratin 9 (CK19) and albumin, was detected in IBDU cultures using phalloidin staining. They had elongated cell bodies with bundles of F-actin terminating at the cell surface typical of fibroblasts (data not shown). Deciliation of cholangiocytes was achieved by incubation with 4 mM of chloral hydrate for 24 h (Masyuk et al., 2006).

### Cultures of cell lines and transfection

Human embryonic kidney (HEK) cells (293T and P1KO) and normal mouse cholangiocytes (NMCs) were cultured in Dulbecco’s modified Eagle’s medium containing 10% heat-inactivated FBS and 1% P/S. *Piezo1*-deficient (P1KO) HEK293T cells were generated using CRISPR/Cas9 nuclease genome engineering (Lukacs et al., 2015). Plasmid transfection (m*Piezo1*iresGFP; m*Piezo1-GFP*; pCMV3-N-*GFPspark*, m*Panx1-flag*) was achieved using Lipofectamine 2000 with 1.2 µg/ml of plasmids.

### Calcium imaging

Cholangiocytes or HEK cells, grown on collagen type I–coated coverslips, were loaded with Fura-2-AM (2 µM) for 45 or 30 min, respectively, at 37°C in Krebs solution (Rodat-Despoix et al., 2013). Fura-2-AM–loaded cells were placed on the stage of an inverted epifluorescence microscope (Olympus IX71) equipped with a 20× UPLSAPO objective and continuously superfused. Fura-2AM was alternately excited at 340 and 380 nm, and the ratios of the resulting images (340 nm/380 nm) were produced every 1 s. The source of excitation light was a xenon arc lamp, and excitation wavelength was selected by a fast excitation filter wheel (Olympus; Illumination Systems MT20). Digital images were sampled at 12-bit resolution by a fast-scan, cooled charge-coupled device (B/W CCD) digital camera (Hamamatsu; Orca-ER). All the images were background-subtracted and controlled by CellˆR software (Olympus). A positive response was defined as a signal at least twice larger than the Δratio deviation determined from a 3-min recording of the standard baseline. The peak ratio value was determined as the difference (Δratio) between the maximum peak value and the baseline during hypotonic shock or drug application. The area under the curve (AUC) above baseline was determined using a trapezoidal method for the duration of drug application. The dose–response curve for Yoda1 was obtained from different experimental sets of coverslips that were prepared from different animals on different days. Dose–response curves were fitted to the data using nonlinear regression. The recording traces in the figures show representative examples of the effect of the drug/test of interest. The histograms/bar charts show pooled data from individual cells (*n*), collected from independent experiments (*N*). The numbers of cells analyzed in each of these independent experiments and the corresponding average responses are presented in Table S4.

### Gene silencing by small interfering RNA (siRNA)

Cholangiocytes at day 1 in vitro (DIV1) were transfected for 7 h with Lipofectamine RNAimax, 10 nM of *Piezo1*-siRNA or scrambled siRNA, and 2 nM of siGLO Red Transfection Indicator without serum and antibiotic. siRNAs are listed in Table S1.

### Immunostaining

Cholangiocytes, HEK293T-P1KO, and HEK293T cells, cultured on (collagen I and poly-D-lysin, respectively) coated glass coverslips, were first washed with PBS and fixed using 4% paraformaldehyde for 10 min, permeabilized in PBS containing 0.1% Triton X-100 for 10 min, blocked with 3% (vol/vol) gelatin for 1 h, and incubated with primary antibodies at 4°C overnight. After washes, cells were incubated with secondary antibodies (1:1,000) at room temperature for 1 h, and nuclei were stained using DAPI for 10 min. Primary and secondary antibodies are listed in Table S5.

### Reverse transcription-PCR

Total RNA was extracted from DIV5 cholangiocytes and NMC with Trizol Reagent according to the manufacturer’s recommendation. RNA concentration was measured using a NanoDrop ND-2000 Spectrophotometer. 4 μg of total RNA were treated with DNaseI, and reverse transcription (RT) reactions with oligo(dT)_20_ primers were performed with the SuperScriptIII First-Strand Synthesis System kit. PCR was performed with *Taq* DNA Polymerase. Sequences of PCR primers are listed in Table S2.

### Real-time RT quantitative PCR (qPCR)

Total RNA extraction and DNase treatment of cholangiocytes were achieved with the NucleoSpin RNA kit according to the manufacturer’s instructions. RT reactions were performed as described above. qPCR reactions were run in duplicate using the Kapa Sybr Fast qPCR kit on an Applied Biosystems 7500 Fast Real-Time PCR thermocycler. Sequences of specific primers are listed in Table S3. The relative quantification (RQ) method was used to compare the relative expression of the *Piezo1* gene between scramble-siRNA (siCtr)– and siPiezo1-transfected cholangiocytes.

### Extracellular ATP measurements

Detection of released ATP was performed using a luciferin/luciferase detection assay using a spectrofluorimeter-luminometer (Tecan Infinite F500). IBDU and HEK cells were cultured in black 96-well Greiner dishes with clear bottoms. ATP secretion was estimated in stimulated and unstimulated cells after a 15-min incubation period with luciferin/luciferase diluted at 1/25 for 5 min. To determine the concentration of ATP, a calibration curve was constructed using known concentrations of ATP (0.02–200 nM).

### Immunoprecipitation from transfected HEK293T-P1KO cells

Protein extraction is detailed in the supplemental text at the end of the PDF. Protein A magnetic beads were coated with 5 µg of rabbit polyclonal anti-GFP antibody or 10 µg of rabbit polyclonal anti-flag antibody or 10 µg of rabbit polyclonal anti-Ki67 antibody (negative control) by rotating at room temperature for 10 min. Cells were added to the beads and rotated for 1 h. Protein complexes were purified by magnetization of the beads and washed with PBS-Tween 0.1% at room temperature. Proteins were eluted from beads by adding Laemmli denaturation sample buffer and incubated 10 min at 70°C.

### Immunoprecipitation from DIV5 cholangiocytes and NMCs

DIV5 cholangiocytes were harvested using TrypLE express and pelleted by centrifugation at 300 *g* for 4 min. Following protein extraction, immunoprecipitation was performed with the Dynabeads coimmunoprecipitation kit according to the manufacturer’s instructions. The primary antibodies, rabbit polyclonal anti-Piezo1 (Thermo Fisher Scientific) and rabbit polyclonal anti-Ki67 (7 µg/mg of beads), were covalently linked to magnetic beads. The extraction buffer was supplemented by adding 150 mM NaCl, 20 mM N-ethylmaleimide, 1.2% Triton X-100, and a cocktail of protease inhibitors without EDTA. Proteins were eluted from beads by adding Laemmli denaturation Sample Buffer and incubated 10 min at 70°C.

### Western blot

Protein samples were denatured by adding 0.1 M dithiothreitol and 3 M urea, and boiling for 10 min at 99°C. Protein samples were loaded in polyacrylamide gel and transferred on a nitrocellulose membrane (GE Healthcare Life Science). Proteins were controlled by Ponceau staining (0.2% wt/vol Ponceau diluted in 5% glacial acetic acid). Membranes were blocked with 5% milk diluted in TBST (Tris-buffered saline Triton) for 1 h at room temperature and probed with primary antibodies overnight at 4°C. The antibodies used are detailed in the supplemental text and Table S5. After washing with TBST, the membranes were incubated for 1 h with horseradish peroxidase (HRP)–coupled goat anti-mouse (1:1,000) or anti-rabbit secondary antibodies. HRP was revealed by chemiluminescence with blotting substrate (1:100), and membranes were scanned on an iBright CL750 imaging system.

### Mass spectrometry on DIV5 cholangiocyte immunocomplexes

See supplemental text at the end of the PDF for further information.

### Chemicals

Stocks of chemicals were reconstructed in either DMSO (1 mM 5-BDBD; 100 mM probenecid [PBC]; 10 mM A-804598; and 10 mM A-740003) or PBS (50 mM ATP; 1 M chloral hydrate; 1 mM gadolinium; 100 U/ml apyrase; 50 mM suramin; 250 mM carbenoxolone [CBX]; and 100 µM GsMTx4) and stored at 4°C or −20°C. Yoda1 was dissolved in 75% DMSO–25% PBS (vol/vol) at 10 mM and stored at −20°C.

### Statistical analysis

Data were expressed as mean ± SEM. Tests for differences between two normally distributed populations were performed using two-tailed Student’s *t* test, and differences in percentages were analyzed by Fisher exact test (GraphPad; Prism 7.00). To compare more than two conditions, data were analyzed by ANOVA and Dunnett’s post-test. P values ≤ 0.05 were considered significant.

### Online supplemental material

**Figure S1.**
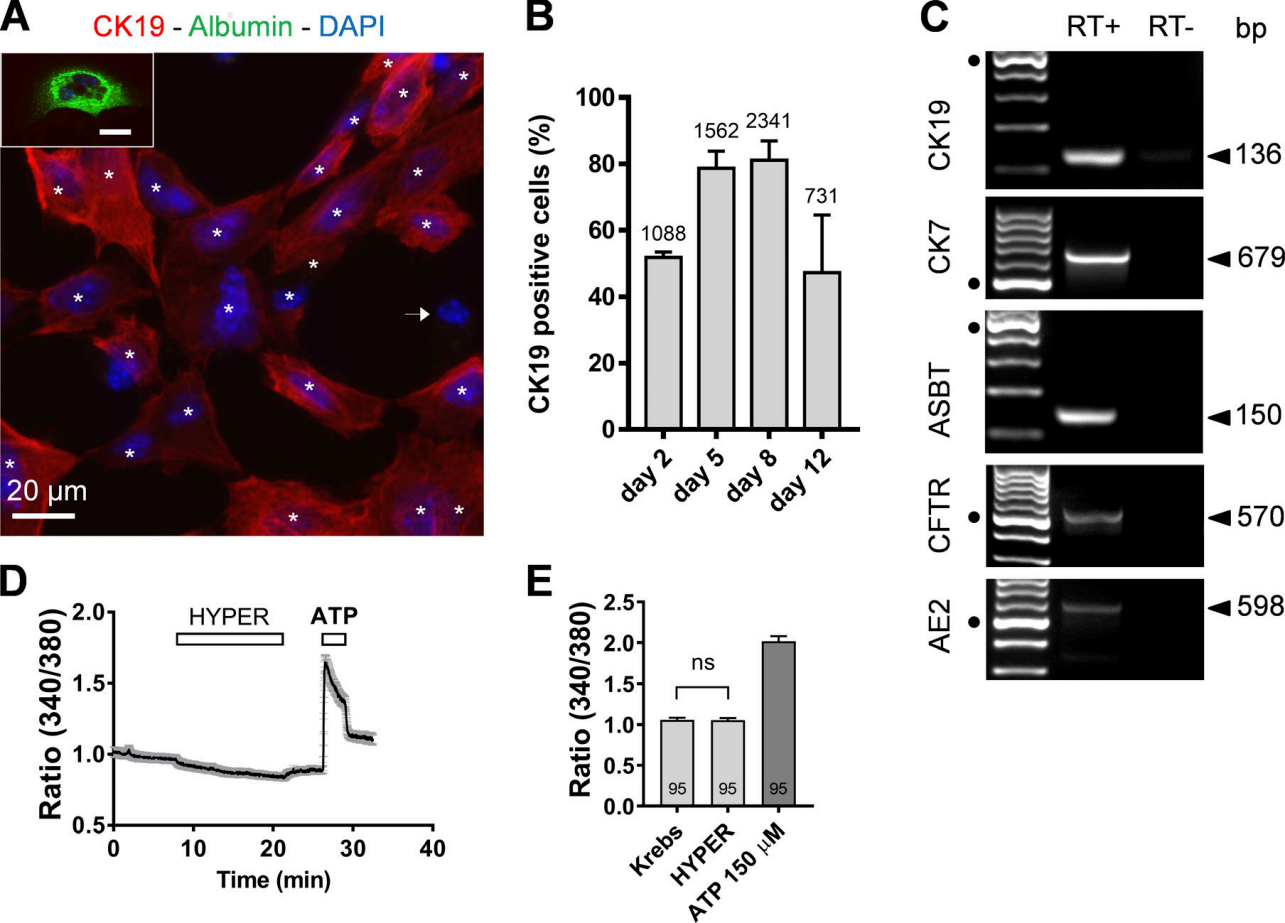
**Mouse cholangiocytes in primary culture are not sensitive to hypertonic stress.** **(A)** Cultured cells from mouse IBDUs were colabeled at DIV5 with an antibody against the cholangiocyte marker CK19 (red) and an antibody against the hepatocyte marker albumin (green). The cell nucleus is stained with DAPI (blue) to allow cell counting. Note that all cells reactive to the CK19 antibody (asterisks) are negative for albumin. The arrow indicates a cell negative to both CK19 and albumin, possibly a fibroblast. Inset: a rare albumin-positive cell seen in mouse IBDU cultures at DIV5. Scale bars, 20 μm. **(B)** Percentage of CK19-positive cells in mouse IBDU cultures. Data normalized to the number of DAPI-positive cells (indicated on each bar). **(C)** IBDU cultures at DIV5 express the typical cholangiocyte markers CK19, CK7, ASBT, CFTR, and AE2a at the mRNA level. Black dots indicate 500-bp DNA marker. Each band is separated by 100 bp. **(D)** Effects of a hypertonic solution (460 mOsmol ⋅ liter^−1^) on DIV5 cholangiocytes. Data points are mean ± SEM (*n* = 26). ATP (150 µM) was applied at the end of the experiment to probe cell viability. **(E)** Ratiometric values determined before (Krebs) and after (HYPER) exposure to the hypertonic solution. Note that ATP (150 µM) caused normal Ca^2+^ mobilization in cells insensitive to the hypertonic solution. ns, not significant (P = 0.5029), paired *t* test.

**Figure S2.**
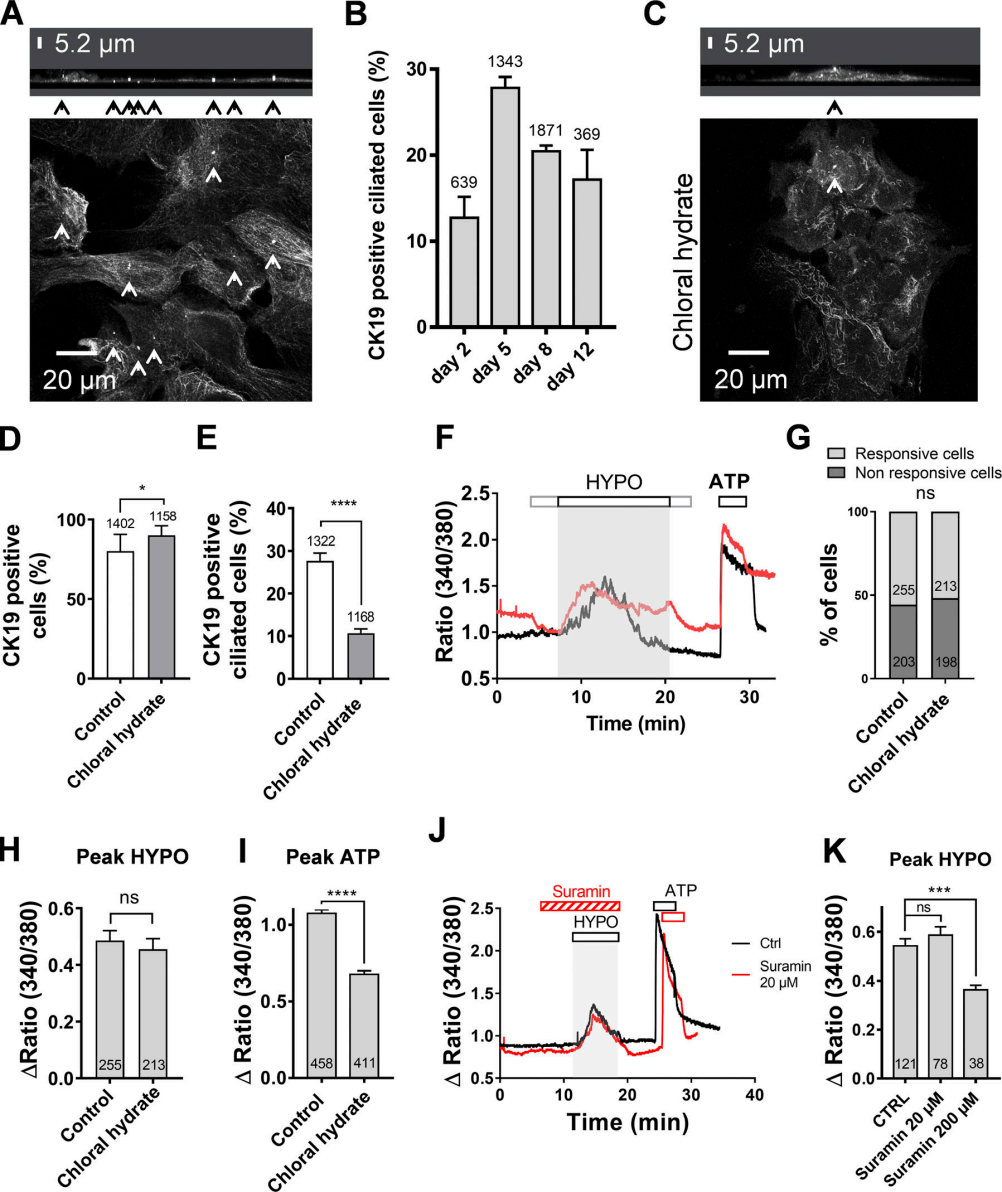
**The primary cilium is not involved in osmosensation.** **(A)** CK19-positive DIV5 cholangiocytes stained with acetylated-tubulin in cultures. Primary cilia are indicated by arrows. The confocal image z-stacks spanned 0.33 µm. Upper: 3-D reconstruction pointing out primary cilia (arrowheads). **(B)** Percentage of ciliated CK19-positive cholangiocytes in mouse IBDU cultures at different times in vitro. The total number of analyzed CK19-positive cells is indicated on each bar. **(C)** Cholangiocytes stained with acetylated-tubulin in cultures treated with chloral hydrate (4 mM, 24 h). **(D)** Percentage of CK19-expressing cells in DIV5 IBDU cultures treated or not with chloral hydrate. *, P = 0.044, Fisher test. **(E)** Chloral hydrate treatment reduces the percentage of ciliated cells among the CK19-expressing cells. ****, P < 0.0001, Fisher test. **(F)** Hypotonic shock-induced Ca^2+^ responses in cholangiocytes pretreated with chloral hydrate (4 mM; red trace) or with the vehicle (black trace). **(G)** Percentage of responsive cholangiocytes in control and chloral hydrate conditions. ns, not significant (P = 0.5378, Fisher test). **(H)** Peak amplitude of hypotonic Ca^2+^ responses in control and chloral hydrate conditions. ns, not significant (P = 0.4445, *t* test). **(I)** Peak Δratio of ATP (150 µM) responses in cholangiocytes from control and chloral hydrate–treated IBDU cultures. ****, P < 0.0001 (unpaired *t* test). **(J)** Hypotonic shock–induced Ca^2+^ responses in cholangiocytes pretreated with suramin (20 µM; red trace) or with the vehicle (black trace). **(K)** Peak amplitude of hypotonic Ca^2+^ responses in control (CTRL) and suramin conditions (20 and 200 µM). ***, P < 0.001 (unpaired *t* test); ns, not significant (P = 0.34; unpaired *t* test). HYPO, hypotonic.

**Figure S3.**
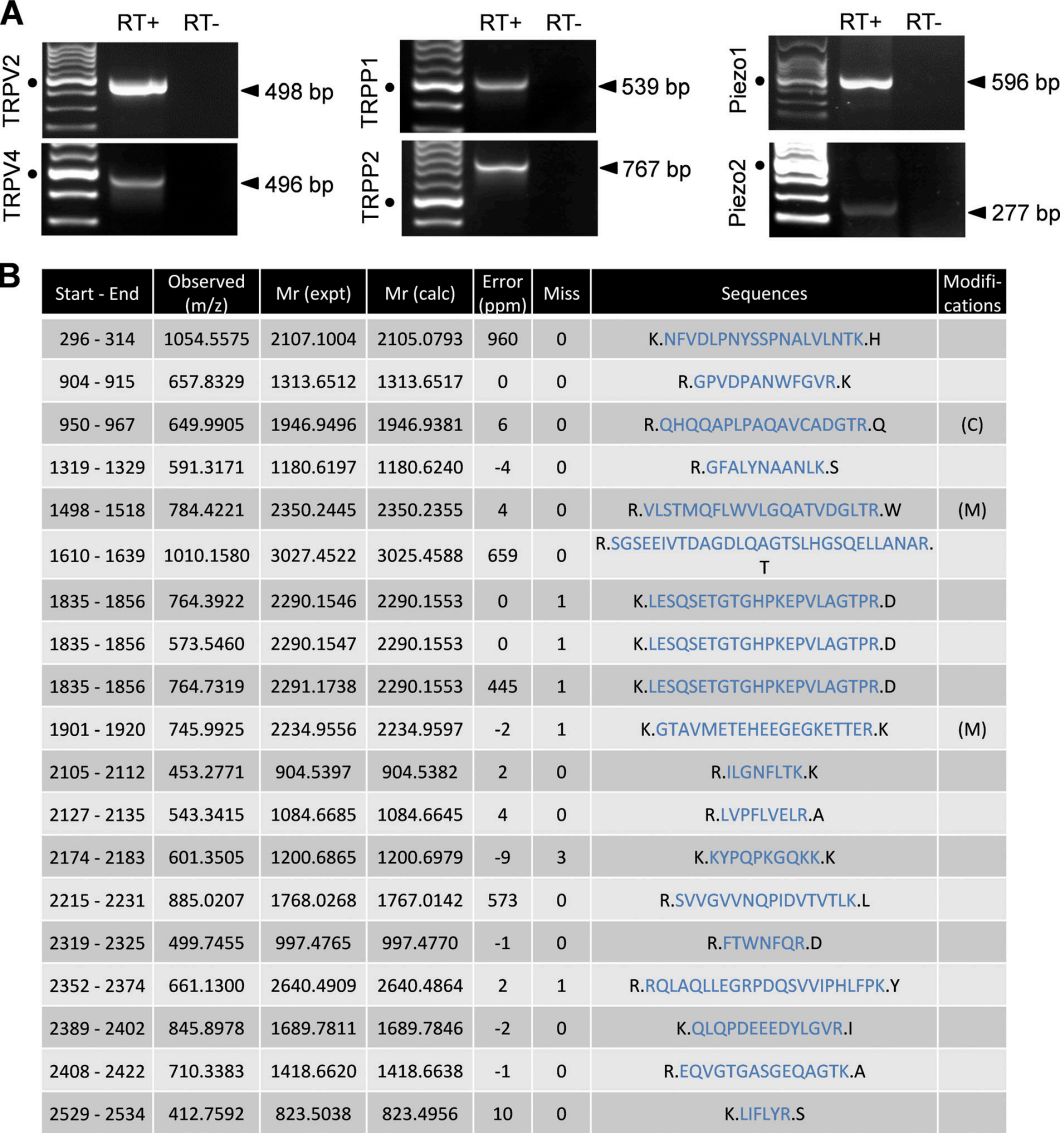
**Expression of putative mechanosensitive channels in cholangiocytes.** **(A)** RT-PCR products from DIV5 cholangiocyte RNA extracts, demonstrating the presence of TRPV2, TRPV4, TRPP1, TRPP2, Piezo1, and Piezo2. Size markers are shown in the left lanes. Black dots indicate the 500-bp DNA marker. **(B)** Identification of Piezo1 by mass spectrometry in cholangiocytes. Piezo1 protein was identified with a sequence coverage of 10%. The identified peptides from mpiezo1 (https://db.systemsbiology.net/sbeams/cgi/PeptideAtlas; E2JF22) are shown in blue, and their position on Piezo1 sequence indicated in column one. Experimental peptide ions m/z (mass to charge ratios), experimental and theoretical peptide masses, experimental error (in ppm), trypsin misscleavages, peptide sequence, and modifications are also indicated. (C), Carbamidomethyl; (M), oxidation.

**Figure S4.**
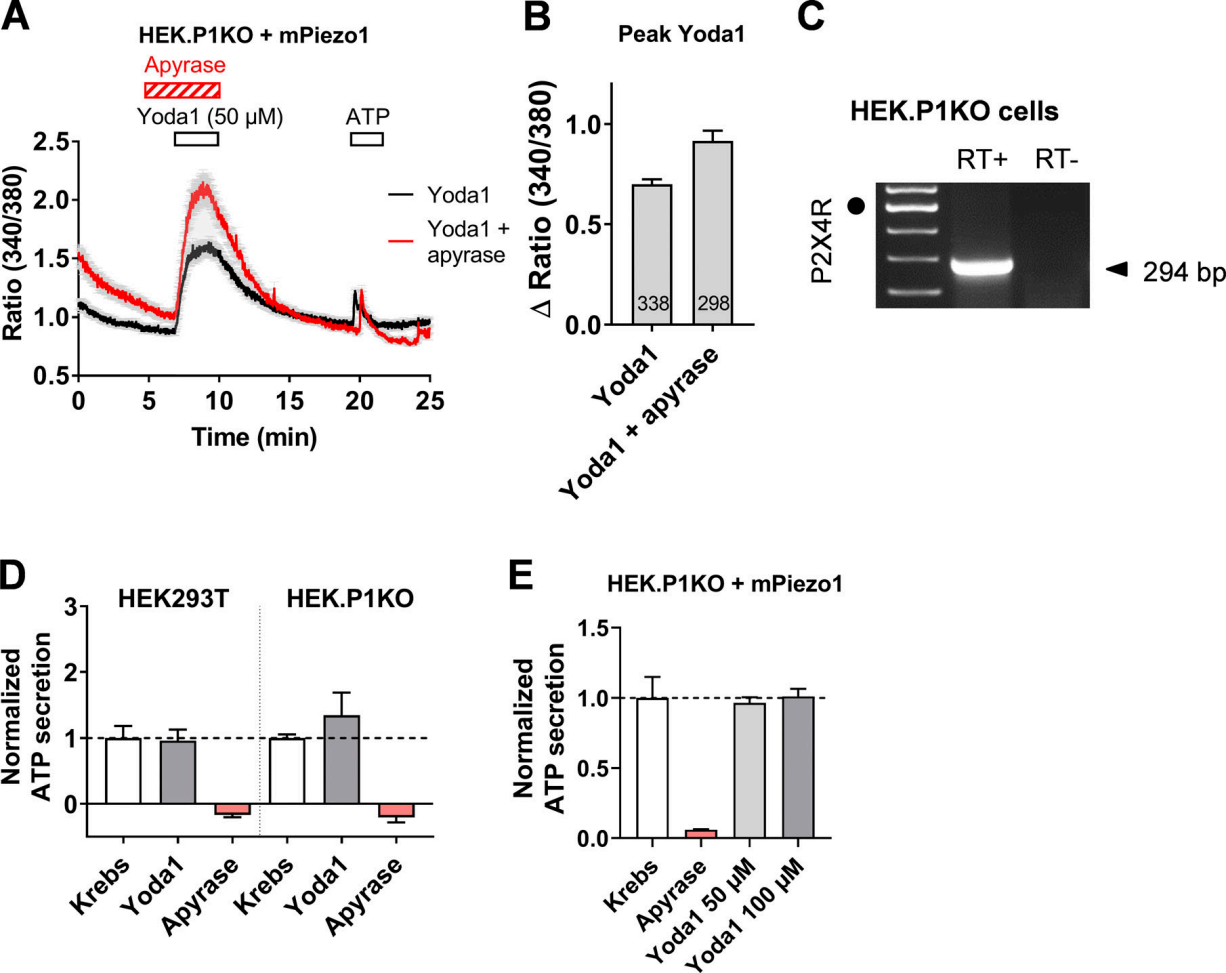
**Yoda1 does not induce ATP secretion by itself.** **(A)** Apyrase did not reduce Yoda1 (50 µM)–induced Ca^2+^ responses in piezo1-GFP–transfected HEKP1KO cells. Data averaged from 115 cells in the two conditions. **(B)** Peak amplitude of Yoda1-induced Ca^2+^ responses in piezo1-GFP–transfected HEKP1KO cells with and without apyrase. **(C)** P2X4R is detected by RT-PCR in native, untransfected HEKP1KO cells. Black dot indicates the 500-bp DNA marker. Data from triplicates. **(D)** Lack of effect of Yoda1 (100 µM) on ATP secretion in HEK293T and HEK293T-P1KO cells. Data are mean ± SEM from triplicates. $\mathrm{Normalized}\;\left[\mathrm{ATP}\right]=\frac{\left(\mathrm{ODkrebs}-\mathrm{ODblank}\right)}{\mathrm{ODkrebs}-\mathrm{ODblank}}.$ **(E)** Lack of effect of Yoda1 on ATP secretion in HEK293T-P1KO cells overexpressing Piezo1-GFP. Data from triplicates.

**Figure S5.**
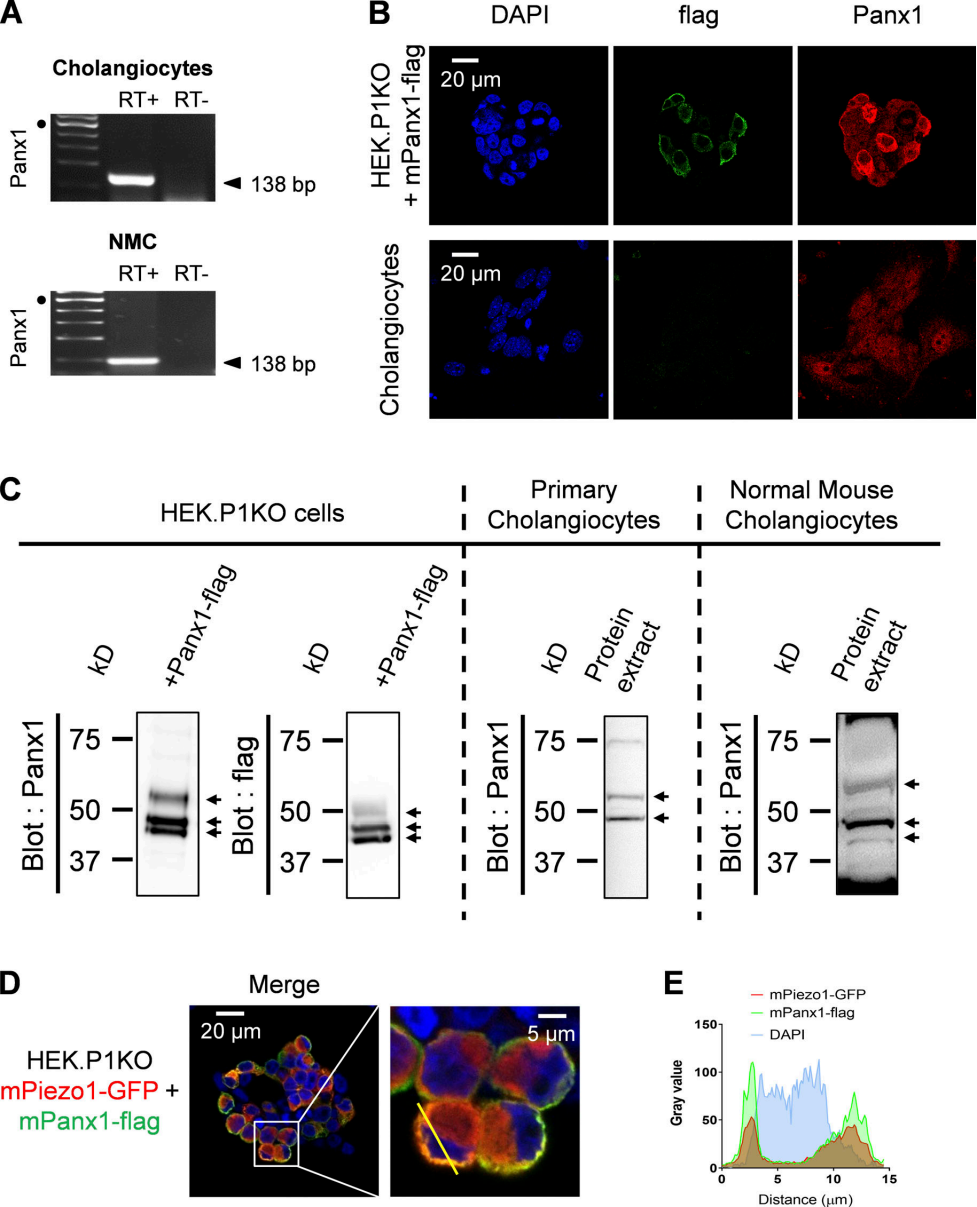
**Panx1 colocalizes with Piezo1 in cholangiocyte plasma membrane.** **(A)** RT-PCR for *Panx1* in DIV5 cholangiocytes and NMCs. Black dots indicate the 500-bp DNA marker. **(B)** Anti-flag and anti-Panx1 immunostainings in DIV5 cholangiocytes (bottom) and in HEK293T-P1KO cells transfected with flag-panx1 cDNA (top). **(C)** Western blot analysis of Panx1 expression in whole cell lysates from HEK293T-P1KO cells expressing Panx1-flag (left), cholangiocytes (middle), and NMCs (right; see also Fig. 7). **(D)** Immunofluorescent staining of Piezo1-GFP and Pannexin1-flag overexpressed in HEK293T-P1KO cells. Cells were labeled using an Alexa 647–coupled anti-flag (shown in green) and a TRITC (tetramethylrhodamine)-coupled anti-GFP (shown in red). **(E)** The line scan illustrates overlap of Piezo1-GFP and Panx1-flag labels at the cell periphery.

**Figure S6.**
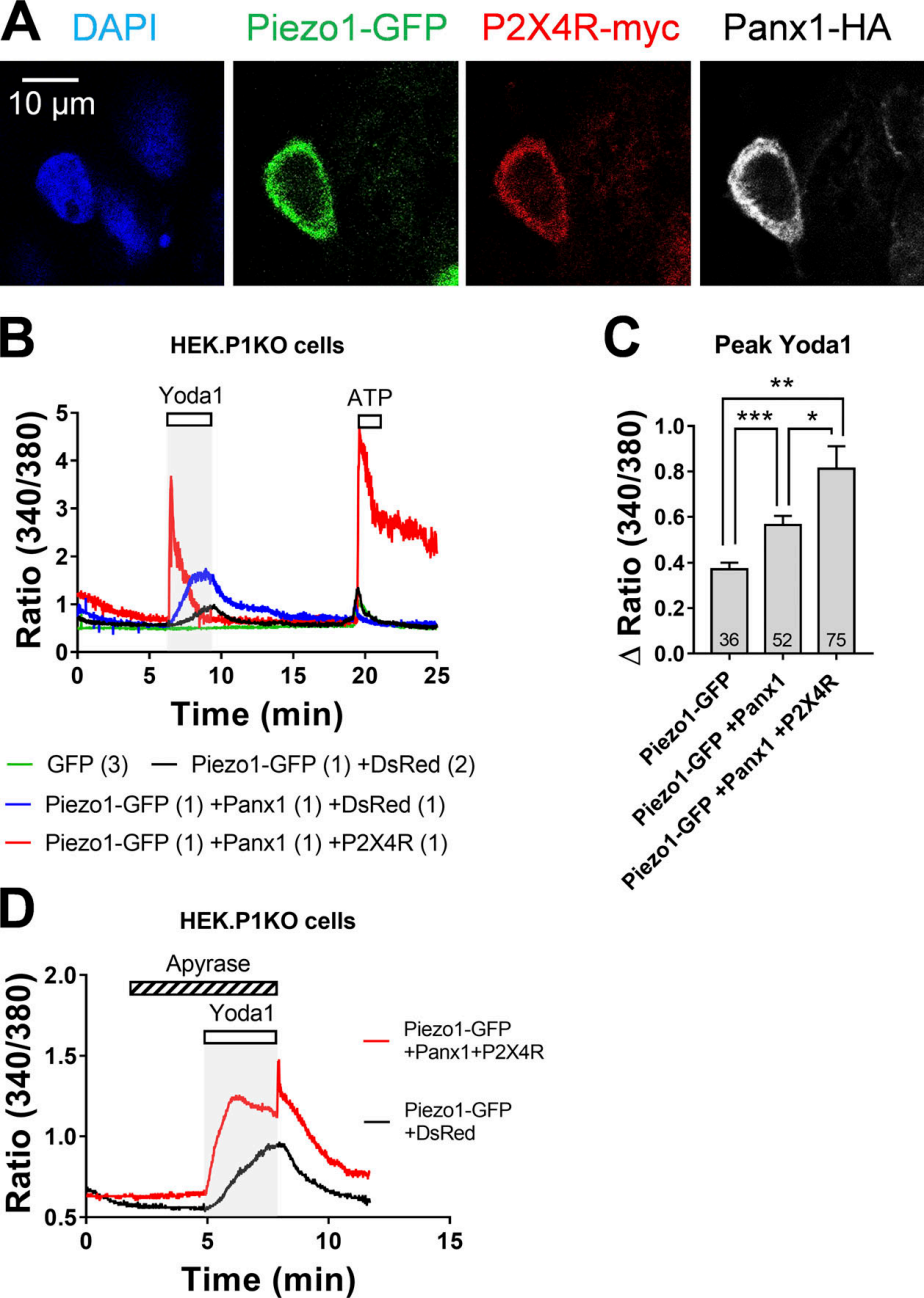
**Reconstitution of Piezo1/Panx1/P2X4R mechanosecretory pathway in HEK293T-P1KO cells.** **(A) **Triple immunofluorescent stainings in a HEK293T-P1KO cell transfected with Piezo1-GFP, Panx1-HA, and P2X4R-myc. **(B)** Ca^2+^ signals in response to Yoda1 (50 µM) in HEK293T-P1KO cells transfected with GFP (green trace), Piezo1-GFP, and DsRed (black trace), Piezo1-GFP, Panx1, and DsRed (blue trace), and Piezo1-GFP, Panx1, and P2X4R (red trace). The ratio of cDNA is indicated in brackets. Note the huge increase in the purinergic response in the cell expressing P2X4R. **(C)** Peak amplitude of Yoda1-induced Ca^2+^ responses in transfected HEK293T-P1KO cells as indicated. *, P = 0.0479; **, P = 0.0029; ***, P = 0.0005 (unpaired *t* test). *n* = 2. **(D)** Yoda1-induced Ca^2+^ responses in HEK293T-P1KO cells expressing Piezo-GFP and DsRed (black trace) or Piezo-GFP, Panx1, and P2X4R (red trace) and treated with apyrase (5 U/ml). Note the overshoot response when apyrase was turned off.

Fig. S1 shows that mouse cholangiocytes in primary culture are not sensitive to hypertonic stress. Fig. S2 shows that the primary cilium is not involved in osmosensation. Fig. S3 shows expression of putative mechanosensitive channels in cholangiocytes. Fig. S4 shows that Yoda1 does not induce ATP secretion by itself. Fig. S5 shows that Panx1 colocalizes with Piezo1 in cholangiocyte plasma membrane. Fig. S6 shows reconstitution of Piezo1/Panx1/P2X4R mechanosecretory pathway in HEK cells. Table S1 shows sequences of siRNA. Table S2 shows sequences of RT-PCR primers. Table S3 shows sequences of qPCR primers. Table S4 shows detailed information depicting the numbers of independent experiments, the number of cells analyzed in each individual experiment, and the corresponding averaged values. Units are not reported and can be found in the figures. Table S5 shows reagents and tools. Supplemental text, available at the end of the PDF, shows further information on qPCR, immunostaining, Western blot, and mass spectrometry protocols.

## Results

### Isolated IBDU cells express cholangiocyte-specific markers

Primary cultures of cholangiocytes were made from mouse IBDUs. Quantitation of the proportion of cells expressing the cholangiocyte-specific marker CK19 revealed that ∼80% of IBDU cells were positive for this marker at DIV5 and DIV8, indicating an enriched population of intrahepatic cholangiocytes (Fig. S1, A and B). Mouse cholangiocytes showed typical polygonal body shape with relatively regular dimensions (Tabibian et al., 2013). The absence of hepatocytes was demonstrated by the lack of staining for albumin, an hepatocyte-specific marker (≤1/1,000 cells; Fig. S1 A). Other cholangiocyte specific markers, including cytokeratin 7 (CK7), cystic fibrosis transmembrane conductance regulator (CFTR), the anion exchanger 2 (AE2), and the apical sodium-dependent bile acid transporter (ASBT), were also detected using RT-PCR (Fig. S1 C).

### Hypotonic stress increases membrane permeability to Ca^2+^ ions

**Figure 1.**
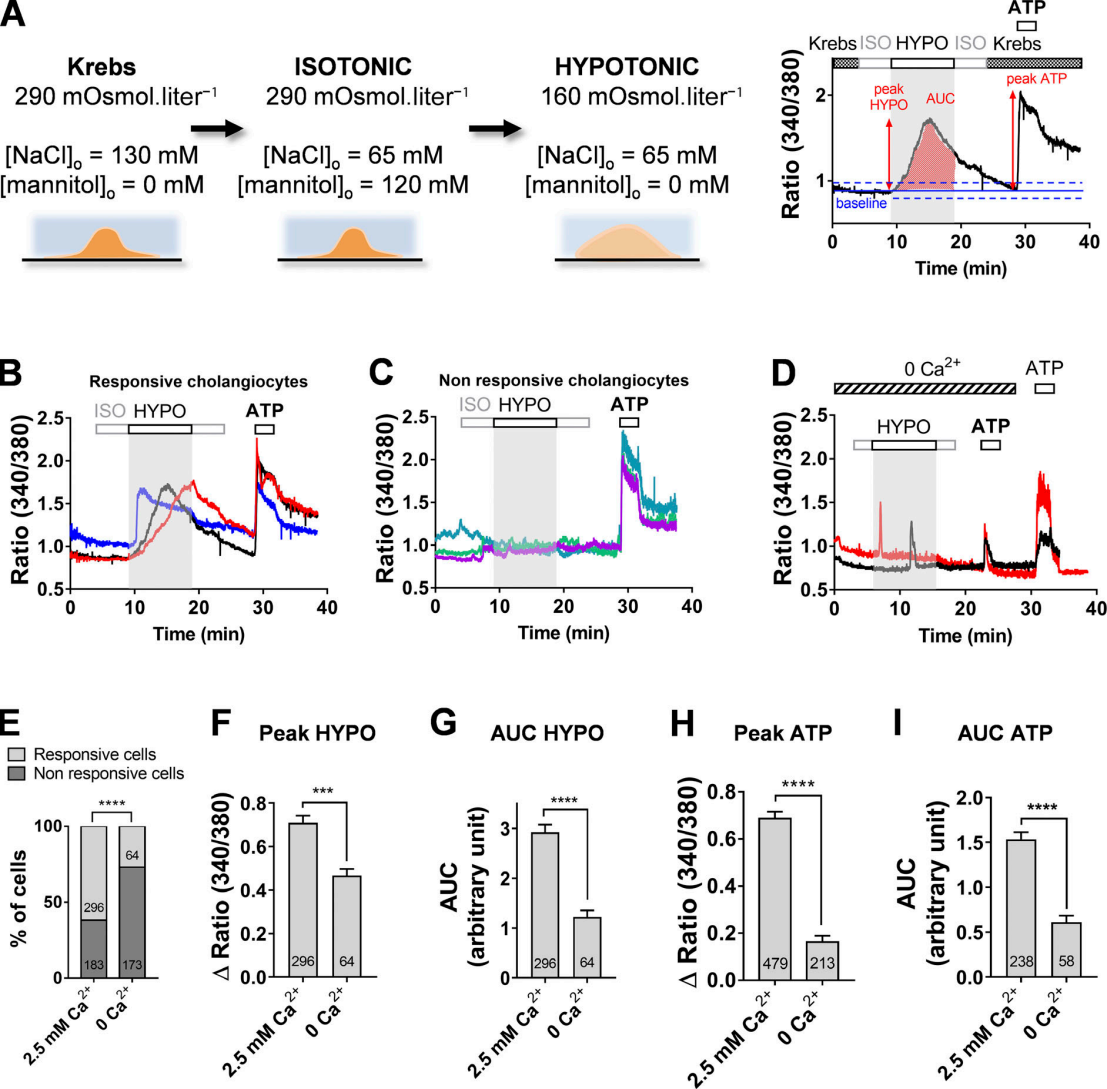
**Hypotonic stress-induced Ca^2+^ signals in mouse cholangiocytes depend on a plasmalemmal calcium-permeable pathway.** **(A)** Left: Composition of the Krebs, isotonic (ISO), and hypotonic (HYPO) solutions. Hypotonic stress is induced by varying the concentration of mannitol, keeping constant the concentration of external ions. Right: Recording of Ca^2+^ signals and determination of the response parameters. Responses to individual stimuli were considered positive (i.e., responsive cells) if deflections exceeded twice the SD (dashed blue line) of the baseline (blue line). AUC (area under the curve) for the time of stimulus application. **(B and C)** Changes in fluorescent signals of the ratiometric calcium indicator Fura-2AM in DIV5 cholangiocytes exposed to hypotonic solution. Representative examples of responsive and nonresponsive cholangiocytes to hypotonic solution are illustrated in B and C, respectively. ATP (150 µM) was applied at the end of the ratio-imaging experiment to monitor cell viability. **(D)** Hypotonic Ca^2+^ signals in cholangiocytes bathed with a Ca^2+^-free external solution. **(E)** Proportion of cholangiocytes showing hypotonic Ca^2+^ responses in the presence or absence of extracellular Ca^2+^. The number of cells analyzed in each condition is indicated. ****, P < 0.0001, Fisher test. **(F and G)** Peak Δratio (340/380 nm; F) and AUC (G) of hypotonic Ca^2+^ responses recorded with and without external Ca^2+^. ***, P = 0.0006; ****, P < 0.0001, unpaired *t* test. **(H and I)** Peak Δratio (340/380 nm; H) and AUC (I) of ATP (150 µM)–induced Ca^2+^ responses with and without external Ca^2+^. ****, P < 0.0001, unpaired *t* test.

We examined the response of cholangiocytes to hypotonic stress (160 mOsm ⋅ liter^−1^) using Fura-2-AM ratio live imaging and measure of Ca^2+^ signals (Fig. 1 A). Cholangiocytes maintained a stable [Ca^2+^]_i_ when in isotonic buffer but showed a slow rise in intracellular Ca^2+^ when bathed with the hypotonic solution (Fig. 1 B). The onset of hypotonic responses followed a variable lag period such that individual cell responses often occurred asynchronously. On average, 74 ± 6% (range, 63–92%) of cholangiocytes were responsive to hypotonic stress (Fig. 1, B and E). Both responsive and nonresponsive cholangiocytes to hypotonic shock showed similar responses to ATP application (150 µM), indicating that nonresponsive cells were healthy (Fig. 1 C). In contrast, cholangiocytes showed no propensity to respond to hypertonic stress (Fig. S1, D and E).

Hypotonic solution–induced Ca^2+^ responses were markedly reduced in Ca^2+^-free solution (Fig. 1 D). Only 27% of cholangiocytes still responded to hypotonic solution under this condition (Fig. 1 E), with a mean peak Δratio reduced by 30% (Fig. 1 F). In addition, most cholangiocytes gave a single Ca^2+^ spike in response to hypotonic stress in Ca^2+^-free conditions (Fig. 1 D). As a result, there was a 60% reduction in the AUC of the hypotonic stress–induced Ca^2+^ responses (Fig. 1 G). Interestingly, purinergic responses also displayed spike-like waveforms in Ca^2+^-free solution, reminiscent of hypotonic Ca^2+^ responses recorded likewise (Fig. 1, D, H, and I). Collectively, these data indicate that hypotonic stress induces slow Ca^2+^ signals in cholangiocytes, which mainly depend on external calcium influx.

### Primary cilium has no role in sensing hypotonic stress

At DIV5, 28% of CK19-positive cells exhibited a primary cilium, as evidenced by acetylated tubulin staining (Fig. S2, A and B). Because primary cilia may sense modifications in osmolarity (Masyuk et al., 2008; Larusso and Masyuk, 2011), we tested whether deletion of the primary cilium could alter hypotonic responses. Incubation of cells with 4 mM chloral hydrate for 24 h resulted in the almost complete loss of primary cilia in CK19-expressing cells (Fig. S2, C and E). This treatment had no deleterious effects on the proportion of CK19-expressing cells (Fig. S2 D). Chloral hydrate incubation had no effect on the proportion of cholangiocytes responding to hypotonic shock, or on the amplitude of Ca^2+^ responses (Fig. S2, F–H). However, we observed a significant reduction of the amplitude of purinergic Ca^2+^ signals (Fig. S2 I), perhaps indicative of the presence of a subset of purinergic receptors located on the cholangiocyte cilium (Larusso and Masyuk, 2011). Collectively, these data indicate that the primary cilium is not required for signaling hypotonic stress.

### Hypotonic stress–induced Ca^2+^ signals depend on ATP release acting primarily on P2X4 receptors

**Figure 2.**
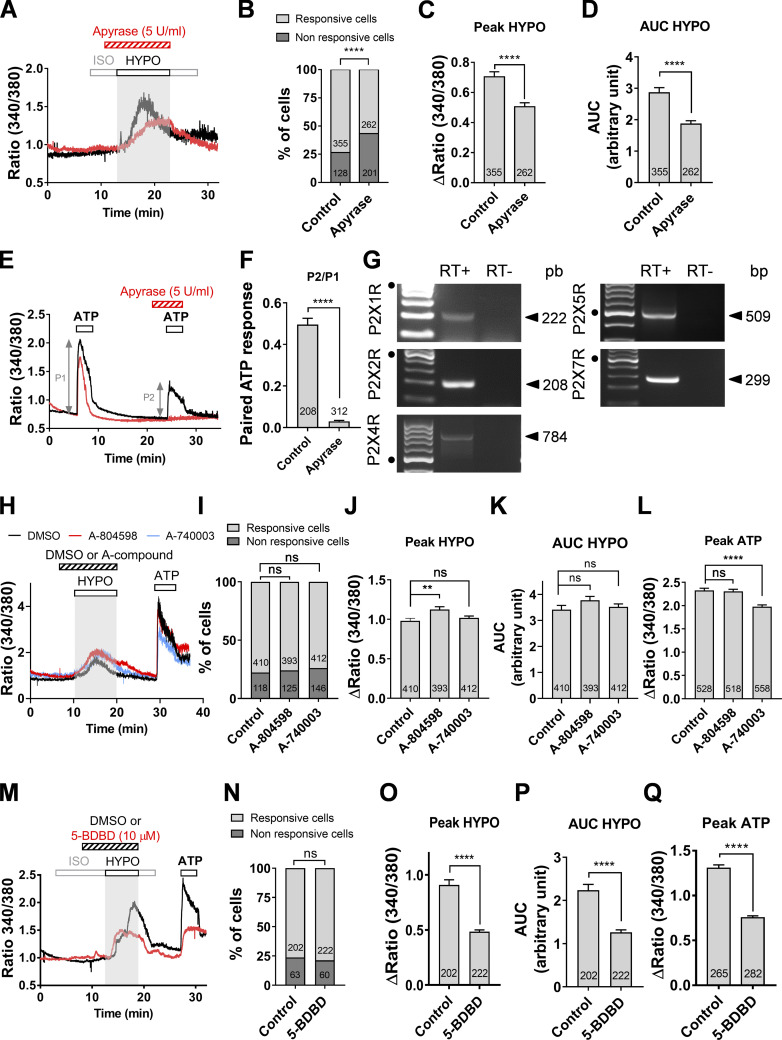
**Hypotonic stress induces ATP secretion and subsequent stimulation of P2X4Rs.** **(A)** Representative changes of ratiometric Ca^2+^ signals in response to hypotonic stress in two DIV5 cholangiocytes treated (red trace) or not (black trace) with apyrase (5 U/ml). **(B)** Percentage of cholangiocytes exhibiting hypotonic Ca^2+^ responses in the presence or absence of apyrase. ****, P < 0.0001 (Fisher test). **(C and D)** Amplitude (C) and AUC (D) of hypotonic Ca^2+^ responses with and without apyrase. ****, P < 0.0001 (unpaired *t* test). **(E)** Paired-pulse ATP stimulation with (red trace) and without (black trace) apyrase. Responses were evoked by two sequential applications of ATP (150 µM) elapsed by 20-min interval. **(F)** Paired pulse depression (P2/P1) of ATP responses examined in the presence or absence of apyrase. ****, P < 0.0001, paired *t* test. **(G)** Cholangiocytes at DIV5 express a variety of P2XR subtypes, including P2X1, P2X2, P2X4, P2X5, and P2X7 receptors at the mRNA level. Black dots indicate 500-bp DNA marker. **(H)** Hypotonic Ca^2+^ responses of DIV5 cholangiocytes treated with A-804598 (0.1 µM, red trace) and A-740003 (0.5 µM, blue trace) or its vehicle (0.1% DMSO, black trace). **(I)** Percentage of cholangiocytes showing hypotonic Ca^2+^ responses in the presence of A-804598 (0.1 µM), A-740003 (0.5 µM), or the vehicle (control). ns, not significant (Fisher test). **(J and K)** Peak Δratio (J) and AUC (K) of hypotonic Ca^2+^ responses recorded with prior incubation with A-804598 (0.1 µM) or A-740003 (0.5 µM). **, P = 0.0027, unpaired *t* test. **(L)** Peak Δratio of ATP responses recorded with prior incubation with A-804598 (0.1 µM) or A-740003 (0.5 µM). ****, P < 0.0001, unpaired *t* test. **(M)** Hypotonic Ca^2+^ responses of DIV5 cholangiocytes treated with 5-BDBD (10 µM, red trace) or its vehicle (0.1% DMSO, black trace). Note the reduced amplitude of both hypotonic and purinergic Ca^2+^ responses. **(N)** Percentage of cholangiocytes showing hypotonic Ca^2+^ responses in the presence of 5-BDBD (10 µM) or its vehicle. ns, not significant (P = 0.5389). **(O and P)** Peak Δratio (O) and AUC (P) of hypotonic Ca^2+^ responses recorded with 5-BDBD (10 µM) or its vehicle. ****, P < 0.0001 (unpaired *t* test). **(Q)** Peak Δratio of ATP responses recorded with prior incubation of 5-BDBD (10 µM). ****, P < 0.0001 (unpaired *t* test). HYPO, hypotonic; ISO, isotonic.

To probe the molecular mechanism that mediates plasmalemmal Ca^2+^ influx under hypotonic conditions, we tested the effects of apyrase, an ATP-diphosphohydrolase that catalyzes the hydrolysis of ATP. Apyrase (5 U/ml) added to hypotonic buffer reduced the proportion of hypotonic solution responding cells from 73.5% to 56.5% (Fig. 2, A and B) and the mean amplitude/AUC of the responses by ∼30% (Fig. 2, C and D). We confirmed that apyrase, at the concentration used, abolished the response to exogenous ATP (150 µM; Fig. 2, E and F).

We found that mouse cholangiocytes at DIV5 express transcripts for P2X1, P2X2, P2X4, P2X5, and P2X7 receptor subtypes (Fig. 2 G). However the nonselective P2R antagonist suramin at 20 µM, a concentration that should affect most subtypes, except P2X7R and P2X4R subtypes (half maximal inhibitory concentration [IC_50_] is >100 µM; North and Jarvis, 2013), had no significant inhibitory effects on hypotonic Ca^2+^ responses (Fig. S2, J and K). Suramin was efficient at relatively high concentrations (200 µM), suggesting the involvement of P2X7R and P2X4R subtypes (Fig. S2, J and K). Therefore, we studied in detail the effects of the selective P2X7R antagonists A804598 and A740003, which have IC_50_ values of 10 and 50 nM for rodent P2X7R, respectively (Donnelly-Roberts et al., 2009). Both A-compounds, used at 10-fold the IC_50_, had no inhibitory effects on hypotonic Ca^2+^ responses (Fig. 2, H–K). Interestingly, they also had modest effects on ATP-induced Ca^2+^ responses, providing further support that P2X7R does not contribute a major component of purinergic signaling (Fig. 2 L).

We further tested the effects of the P2X4R antagonist 5-BDBD, which exhibits no significant antagonist effects at other P2XRs (Coddou et al., 2019). 5-BDBD reduced by ∼50% both the amplitude and AUC of hypotonic responses (Fig. 2, M–P). Interestingly, 5-BDBD also inhibited Ca^2+^ responses evoked by exogenous ATP by the same amount (Fig. 2 Q). Altogether, these findings suggest that the P2X4R contributes to the ATP-dependent hypotonic calcium response.

### The mechanosensitive ion channel Piezo1 is functionally active in mouse cholangiocytes

**Figure 3.**
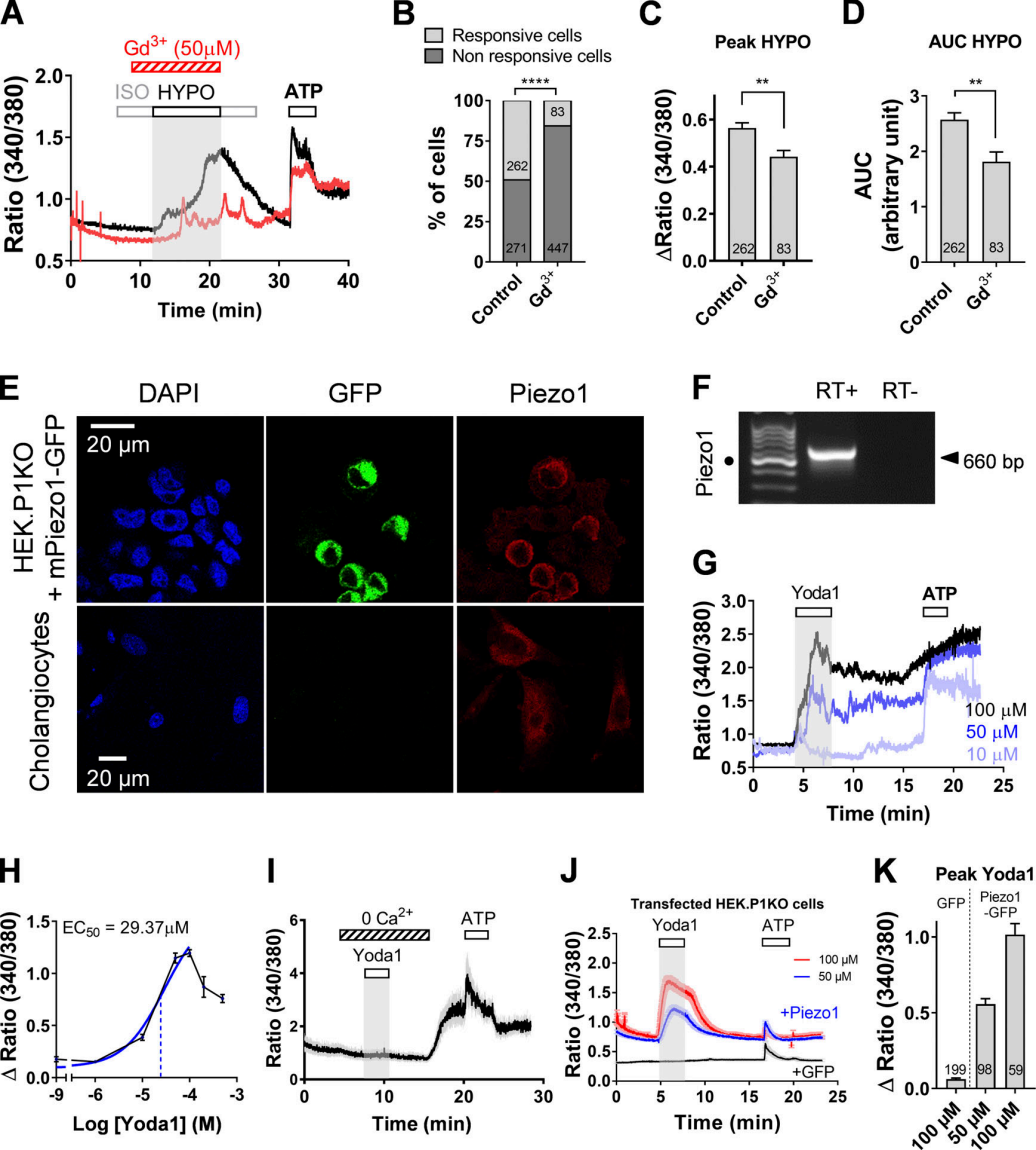
**Piezo1 is expressed and functional in mouse cholangiocytes.** **(A)** Hypotonic Ca^2+^ responses of DIV5 cholangiocytes treated (red trace) or not (black trace) with Gd^3+^ (50 µM). Note that Gd^3+^ reduces the amplitude of the hypotonic Ca^2+^ response and converts the response into spike-like signal. **(B)** Percentage of cholangiocytes showing hypotonic Ca^2+^ responses in the presence of Gd^3+^ (50 µM). ****, P < 0.0001 (Fisher test).** (C and D)** Peak Δratio (C) and AUC (D) of hypotonic Ca^2+^ responses recorded in the presence of Gd^3+^ (50 µM). **, P = 0.0042 (C) and P = 0.0018 (D; unpaired *t* test). **(E)** Immunostaining for Piezo1 in HEK293T-P1KO cells transfected with *GFP-mpiezo1* cDNA (top panels) and in DIV5 mouse cholangiocytes (bottom). **(F)** RT-PCR for *mpiezo1* in DIV5 cholangiocytes. Black dot indicates 500-bp DNA marker. **(G)** Ca^2+^ signals of DIV5 cholangiocytes in response to increasing concentrations (10, 50, and 100 µM) of Yoda1. **(H)** Concentration-response profile for Yoda1 in mouse DIV5 cholangiocytes, yielding apparent EC_50_ of 29.37 ± 1.25 µM (*n* = 99–278). **(I)** Effect of Yoda1 (50 µM) in the absence of external calcium. Data averaged over 84 individual cholangiocytes. **(J)** Ca^2+^ signals in response to Yoda1 (50 and 100 µM) in HEK293T-P1KO cells transfected with mPiezo1iresGFP or pGFP cDNAs (100 µM Yoda1). Traces are mean ± SEM of 60 individual responses per condition. **(K)** Peak Δratio of Yoda1 responses in HEK293T-P1KO cells transfected with mPiezo1iresGFP or pGFP cDNAs. HYPO, hypotonic; ISO, isotonic.

As a first step toward identifying the plasmalemmal channel(s) that senses hypoosmotic challenge and primes ATP release, we applied Gd^3+^ (50 µM), a broad-spectrum inhibitor of stretch-activated ion channels. Under Gd^3+^ application, 85% of cholangiocytes were unresponsive to hypotonic shock (Fig. 3, A and B). The few remaining responsive cells had reduced peak responses with features reminiscent of those seen in Ca^2+^-free solution (Fig. 3, A, C, and D; compare with Fig. 1 D).

Multiple putative mechanosensitive ion channels are known to be expressed in cholangiocytes; however, with the notable exception of TRPV4, there is little evidence supporting any of these as a component of the osmosensitive transduction complex. With the use of RT-PCR assay from DIV5 primary cultures, we detected RNA for the vanilloid subtypes TRPV2 and TRPV4, the members of the polycystin-like groups TRPP1 and TRPP2 (Delmas, 2004), and the mechanosensitive ion channel Piezo1 (Coste et al., 2010; Fig. 3 F and Fig. S3 A). *mPiezo1* was detected with different sets of primers (see Materials and methods and Table S2), whereas Piezo2 was barely detectable (Fig. S3 A). Immunostaining using a polyclonal Piezo1 antibody (Thermo Fisher Scientific; see Materials and methods and Table S5) further showed labeling of cholangiocytes (Fig. 3 E). To verify that the antibody interacts with the intended target, we transfected *mpiezo-GFP* in HEK293T-P1KO cells. Strong immunoreactivity for Piezo1 was found in HEK293T-P1KO cells that have been effectively transfected (Fig. 3 E). Unfortunately, no specific banding was observed in Western blots from *mpiezo1*-transfected HEK293T-P1KO cells with this anti-Piezo1 antibody. The same holds for the five other commercially available antibodies tested (see Materials and methods). However, using immunoprecipitation with the Thermo Fisher Scientific anti-Piezo1 antibody coupled to mass spectrometry, we identified mPiezo1 peptide sequences from lysates of DIV5 cholangiocytes (Fig. S3 B).

To stimulate Piezo1 channels, we used the Piezo1 agonist, chemical compound Yoda1, which is selective for Piezo1 over Piezo2 (Botello-Smith et al., 2019; Syeda et al., 2015). Bath application of Yoda1 in DIV5 cholangiocytes evoked robust Ca^2+^ signals (Fig. 3 G). Approximate half-maximal effective concentration (EC_50_) for Yoda1 in cholangiocytes was 29.4 ± 1.25 µM (Fig. 3 H), which compares well with EC_50_ of 17.1 and 26.6 µM for murine and *hpiezo1*-transfected HEK293T cells, respectively (Syeda et al., 2015). Yoda1 signal was dependent on extracellular Ca^2+^ in cholangiocytes (Fig. 3 I) and exclusively related to the expression of Piezo1, since Yoda1 had no effects on HEK293T-P1KO cells (Fig. 3, J and K).

### Piezo1 contributes to hypotonic stress–induced Ca^2+^ signals in cholangiocytes

**Figure 4.**
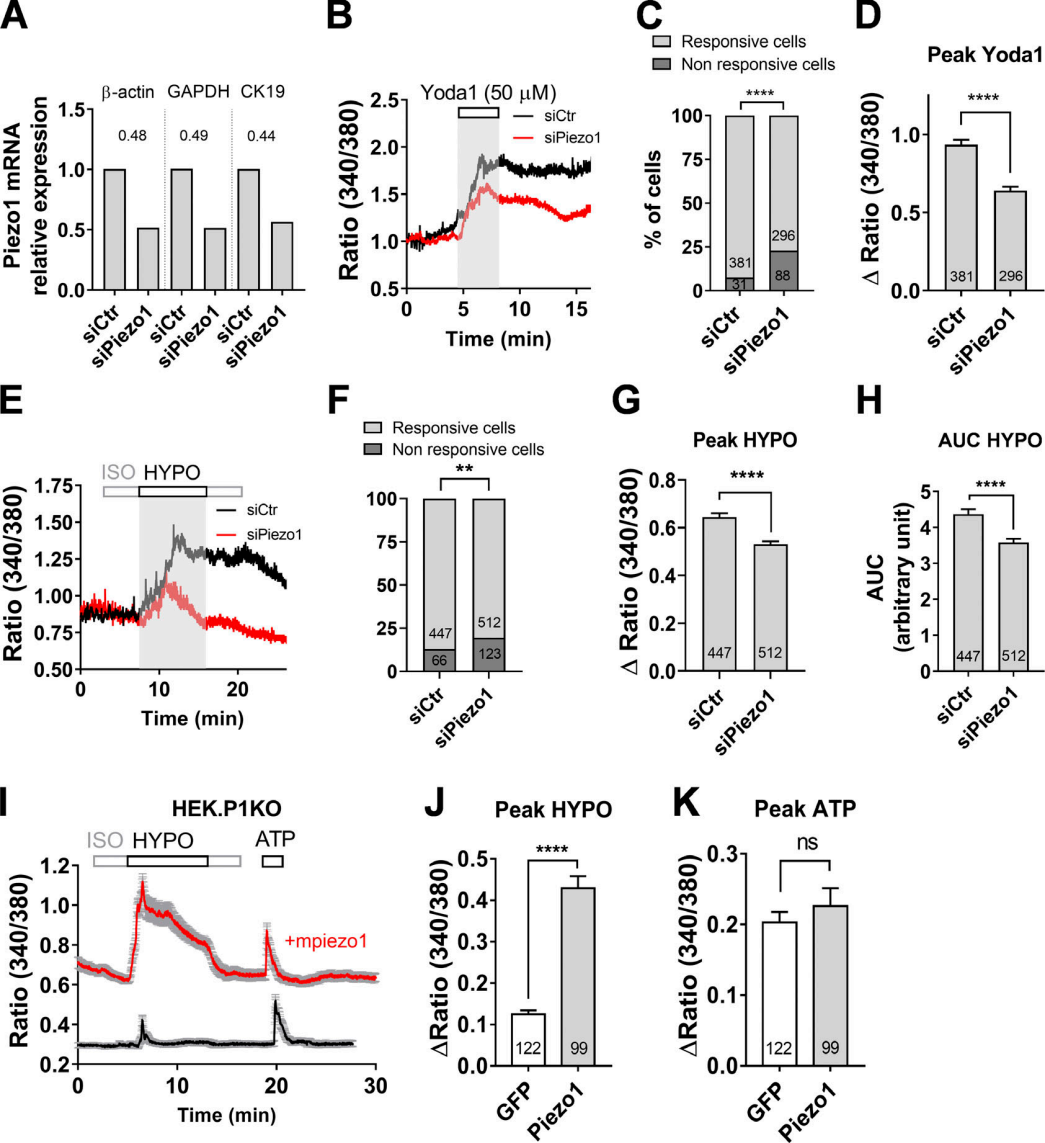
**Piezo1 contributes to hypotonic stress-induced Ca^2+^ signals in cholangiocytes.** **(A)** Quantitative real-time PCR analysis of the efficiency of *piezo1*-siRNA. Relative levels of *piezo1* mRNA in cholangiocytes normalized to the housekeeping genes β-actin, GAPDH, and CK19. Data are presented as mean ± SEM based on cholangiocytes transfected with *piezo1* or siCtr. **(B)** Yoda1-induced Ca^2+^ responses recorded in DIV5 cholangiocytes 96 h after transfection with siCtr (black trace) or siPiezo1 (red trace). **(C and D)** Percentage of cholangiocytes showing Yoda1-induced Ca^2+^ responses (C) and corresponding peak amplitude (D) after transfection with siCTR or siPiezo1. ****, P < 0.0001, Fisher test (C) and unpaired *t* test (D). **(E)** Hypotonic Ca^2+^ responses recorded in DIV5 cholangiocytes 96 h after transfection with siCtr (black trace) or siPiezo1 (red trace). **(F)** Percentage of cholangiocytes showing hypotonic Ca^2+^ responses 96 h after transfection with siCtr or siPiezo1. **, P = 0.0031, Fisher test. **(G and H)** Peak Δratio (G) and AUC (H) of hypotonic Ca^2+^ responses recorded after 96 h transfection with siCtr or siPiezo1. ****, P < 0.0001, unpaired *t* test. **(I)** Hypotonic Ca^2+^ responses in HEK293T-P1KO cells transfected with GFP cDNA (black trace) or mPiezo1iresGFP cDNA (red trace). Data shown are mean ± SEM for 52 and 36 cells, respectively. **(J and K)** Peak Δratio of hypotonic responses (J) and ATP (150 µM) responses (K) in HEK293T-P1KO cells transfected with GFP or mPiezo1iresGFP cDNAs. ****, P < 0.0001; ns, P = 0.4124, unpaired *t* test. HYPO, hypotonic.

To test whether Piezo1 contributes to hypotonicity-induced Ca^2+^ responses, we inhibited endogenous Piezo1 by using specific *piezo1*-siRNAs targeting four distinct sequences of *piezo1* mRNA. Quantification of mRNA transcripts and data normalization were carried out by qPCR against the invariant endogenous housekeeping genes GAPDH, β-actin, and CK19. As shown in Fig. 4 A, *piezo1* mRNA level decreased twofold in *piezo1*-siRNA–transfected cholangiocytes compared with those transfected with scrambled siRNAs. Accordingly, *Piezo1*-siRNA significantly reduced the responses of cholangiocytes to Yoda1 exposure (Fig. 4, B–D). *Piezo1*-siRNA also reduced the proportion of cholangiocytes responding to hypotonic stress and the peak/AUC of the responses (Fig. 4, E–H). Collectively, these data suggest that Piezo1 activation contributes to hypotonic calcium responses. Consistent with a role in osmosensation, overexpression of mPiezo1 promoted hypotonic responses in characteristically unresponsive HEK293T-P1KO cells to hypotonic stimulation (Fig. 4, I–K).

### Hypotonic stress–induced activation of Piezo1 promotes ATP release

**Figure 5.**
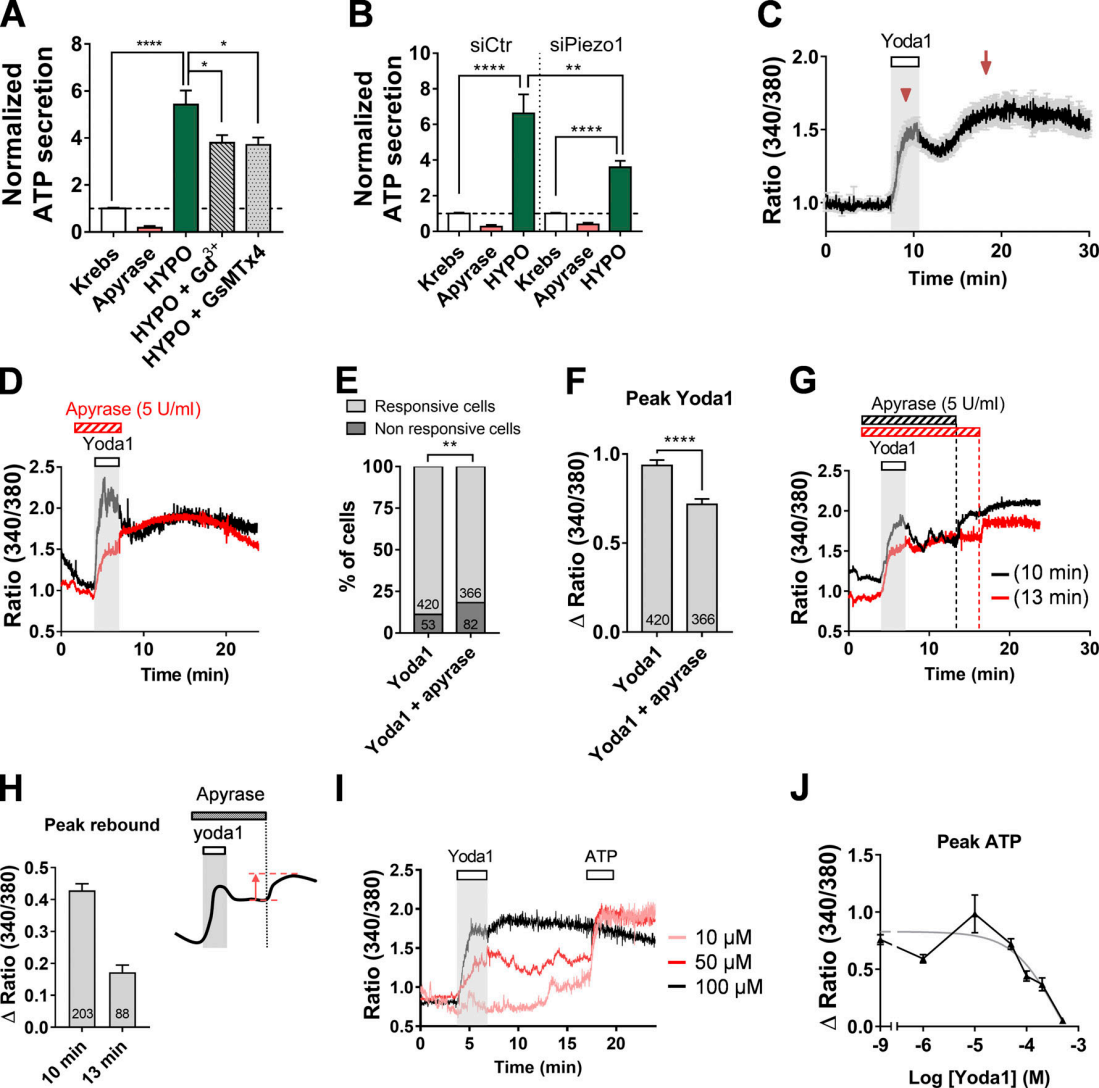
**Piezo1 contributes to hypotonic shock-induced ATP secretion.** **(A)** ATP release induced by hypotonic shock in DIV5 cholangiocytes in the presence of Gd^3+^ (100 µM) or GsMTx4 (5 µM). Data represent means ± SEM for five or six separate experiments. *, P = 0.0176; **, P = 0.0012; ****, P < 0.0001; two-way ANOVA followed by Dunnett’s multiple comparison test. **(B)** ATP release induced by hypotonic shock in DIV5 cholangiocytes after 96 h transfection with siCtr or siPiezo1. Data from nine independent experiments normalized to nonstimulated sister cultures. ****, P < 0.0001, one-way ANOVA, Dunnett's multiple comparison test; **, P = 0.01, unpaired *t* test. **(C)** Yoda1 (50 µM)–induced Ca^2+^ response averaged (± SEM) over 110 individual DIV5 cholangiocytes. Note the biphasic shape of the Ca^2+^ response with an initial Ca^2+^ rise (arrowhead) followed by a delayed component (arrow). **(D)** Representative Yoda1 (50 µM)–induced Ca^2+^ responses in the absence (black trace) or presence of apyrase (5 U/ml; red trace). **(E and F) **Proportion of cholangiocytes showing Yoda1-induced Ca^2+^ responses in the presence or absence of apyrase (E) and corresponding amplitude of the initial component (F). **, P = 0.028, Fisher test; ****, P < 0.0001, unpaired *t* test. **(G)** Representative Yoda1 (50 µM)–induced Ca^2+^ responses in the presence of apyrase (5 U/ml) applied for different durations (10 min, black trace; 13 min, red trace) after Yoda1 washout. Note the occurrence of a rebound Ca^2+^ component at the apyrase offset. **(H)** Amplitude of Ca^2+^ rebound occurring at the offset of 10 and 13 min apyrase exposure. Experiments as in G (*n* = 3).** (I and J)** Amplitude of ATP responses as a function of prior exposure to Yoda1 at increasing concentrations. ATP (150 µM) was applied 10 min after the offset of Yoda1 application (I). Each data point in J shows mean ± SEM from 54–278 cells. HYPO, hypotonic.

Whether ATP release induced by hypotonic stimulation was subsequent to Piezo1 activation was first assessed by pharmacological assays. Using the luciferase-ATP bioluminescent detection assay, we detected a sixfold increase in ATP release, from 2.85 ± 0.43 to 17.58 ± 2.28 nM (*n* = 10), following hypotonic stimulation (Fig. 5 A). ATP release was inhibited by ∼30% by Gd^3+^ (100 µM) and GsMTx4 (5 µM), a peptide from tarantula venom effective against Piezo1 at low micromolar concentrations (Suchyna et al., 2000; Fig. 5 A). Because none of these inhibitors are piezo-specific, we tested whether *piezo1*-siRNAs could inhibit hypotonic stress–induced ATP release. ATP secretion was decreased by 46% in *piezo1*-siRNA–treated cholangiocytes compared with control-siRNA–treated cells (Fig. 5 B).

To further investigate Piezo1-dependent ATP release, we examined Ca^2+^ mobilization upon exposure to Yoda1. One trait of Yoda1 responses was their biphasic shape composed of an initial rise in [Ca^2+^]_i_, concurrent with Yoda1 application, followed by a delayed, slow [Ca^2+^]_i_ increase (Fig. 5 C). The delayed “rebound” response often outperformed the initial Yoda1 response. Importantly, when apyrase was applied concurrently with Yoda1, only the initial phase was significantly diminished (Fig. 5, D–F). However, the delayed response was impaired when apyrase was maintained beyond Yoda1 exposure, but resumed as soon as apyrase was washed out (Fig. 5 G). Consequently, there was a clear relationship between the occurrence of the delayed phase and the duration of apyrase application (Fig. 5 H). Another manifestation of sustained ATP release induced by Yoda1 was the observation that cholangiocytes became refractory to ATP stimulation following exposure to Yoda1 (Fig. 5 I). Consequently, the amplitude of ATP responses was inversely correlated with the concentration of Yoda1 (Fig. 5 J). Collectively, these data indicate that Piezo1 activation by Yoda1 promotes long-lasting ATP release.

### Piezo1 is not the ATP secretion pathway

Does Piezo1 by itself qualify as a permeation pathway for ATP? To answer this question, we took advantage of the fact that Yoda1 did not generate Ca^2+^ responses in HEK293T-P1KO cells. However, Yoda1 caused large Ca^2+^ signals in HEK293T-P1KO cells overexpressing mPiezo1 (see Fig. 3 J). Importantly, in these cells, Yoda1-induced Ca^2+^ signals were not inhibited by apyrase (Fig. S4, A and B), suggesting that ATP does not amplify Yoda1-induced Ca^2+^ responses in mPiezo1-expressing HEK293T-P1KO cells, although they express P2X4R (Fig. S4 C). As a corollary, no secretion of ATP was detected in response to Yoda1 application in HEK293T cells, HEK293T-P1KO cells, and HEK293T-P1KO cells overexpressing mPiezo1 (Fig. S4, D and E). These observations are all suggestive of a channel-mediated ATP release mechanism distinct from Piezo1.

### Piezo1-driven activation of Panx1 mediates ATP release

**Figure 6.**
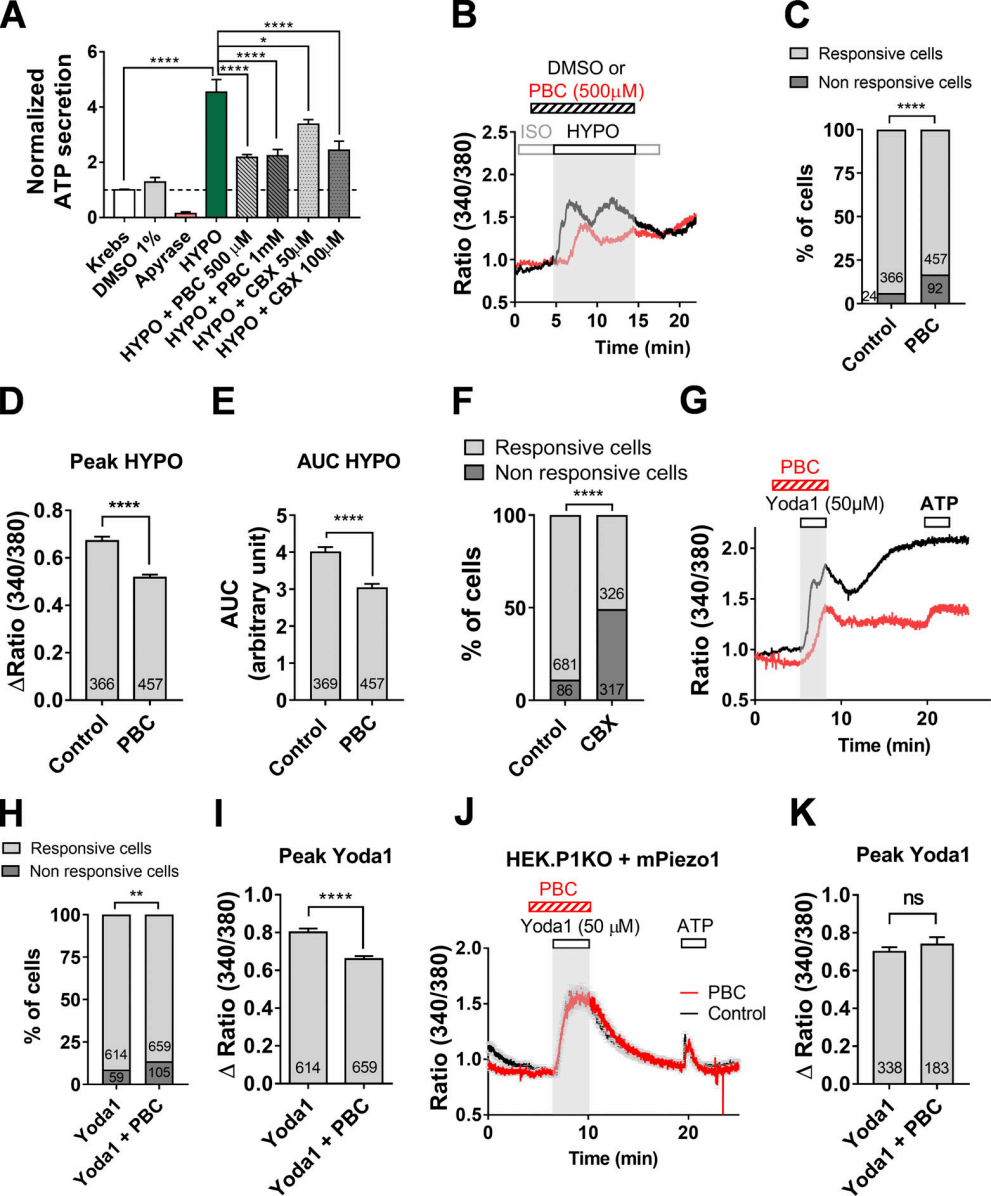
**Pannexin1 contributes to hypotonic stress-induced ATP release.** **(A)** ATP release induced by hypotonic stress in DIV5 cholangiocytes in the presence of PBC (500 µM, 1 mM) and CBX (50–100 µM). Data represent mean ± SEM for four to nine separate experiments. *, P < 0.026; ****, P < 0.0001 (ANOVA followed by Dunnett’s multiple comparison test). **(B)** Representative hypotonic Ca^2+^ responses in the presence (red trace) or absence (DMSO, black trace) of PBC (500 µM). **(C)** Percentage of cholangiocytes showing hypotonic Ca^2+^ responses in the presence or absence of PBC. ****, P < 0.0001 (Fisher test). **(D and E)** Peak Δratio (D) and AUC (E) of hypotonic Ca^2+^ responses recorded in the presence of PBC. ****, P < 0.0001 (unpaired *t* test). **(F)** Proportion of cholangiocytes showing hypotonic-induced Ca^2+^ responses in the presence or not of CBX. ****, P < 0.0001 (unpaired *t* test). **(G)** Representative Yoda1 (50 µM)–induced Ca^2+^ responses in the absence (black trace) or presence of PBC (500 µM, red trace). **(H and I)** Proportion of cholangiocytes showing Yoda1-induced Ca^2+^ responses (H) and corresponding peak amplitude (I) in the presence or not of PBC. **, P = 0.0035 (Fisher test; H); ****, P < 0.0001 (unpaired *t* test; I). **(J)** Yoda1 (50 µM)–induced Ca^2+^ responses in HEK293T-P1KO cells transfected with mPiezo1iresGFP cDNA in the presence (red trace, *n* = 123) or not (black trace, *n* = 115) of PBC (500 µM). **(K)** Peak Δratio of Yoda1-induced Ca^2+^ responses recorded in HEK293T-P1KO cells transfected with mPiezo1iresGFP cDNA with or without PBC. ns, P = 0.386 (unpaired *t* test). HYPO, hypotonic.

We found strong expression of Panx1 in DIV5 cholangiocytes using RT-PCR, immunocytochemistry, and Western blots (Fig. S5, A–C). We investigated the role of Panx1 using CBX and PBC, which are well-known to inhibit Panx1-mediated ATP release in a variety of systems (Locovei et al., 2006; Silverman et al., 2008). Pretreatment of cholangiocytes with CBX or PBC decreased the secretion of ATP induced by hypotonic stimulation by ∼50% (Fig. 6 A). PBC also decreased the proportion of cholangiocytes that responded to hypotonic stress along with the amplitude of the Ca^2+^ responses (Fig. 6, B–E). Likewise, CBX also decreased significantly the proportion of cholangiocytes responsive to hypotonic stimulation from 89% in control to 51% with CBX (Fig. 6 F). In addition, PBC reduced Yoda1-induced Ca^2+^ responses, acting on both the initial and the delayed components of Yoda1 responses (Fig. 6, G–I). Because PBC may have off-targets, we confirmed that PBC had no direct effects on Piezo1 since Yoda1-induced responses in HEK293T-P1KO cells overexpressing Piezo1 were not impaired (Fig. 6, J and K). Collectively, these findings identify a role for Panx1 in ATP secretion secondary to hypotonic stimulation of Piezo1.

### Piezo1 and Panx1 can assemble within a protein complex

**Figure 7.**
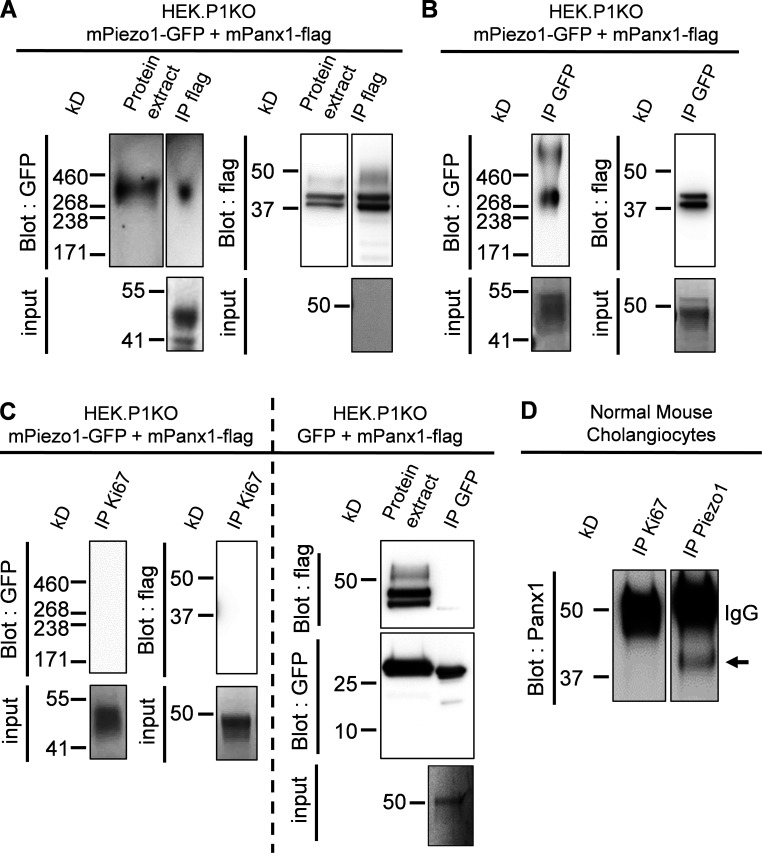
**Identification of Panx1 as an interacting protein of Piezo1.** **(A and B)** Immunoprecipitates from HEK293T-P1KO cells cotransfected with *Piezo1-GFP* and *Panx1-flag* cDNAs. Protein interaction was analyzed by immunoprecipitation (IP) using anti-flag (A) and anti-GFP (B) antibodies, followed by immunoblots with the indicated antibodies. Six independent experiments. Inputs: Blot membranes were stained with Ponceau red to verify appropriate protein transfer and the presence of the heavy chain of antibodies used for IPs. **(C)** Pull-down was absent using an anti-Ki67 antibody as control for isotype IgG (left). Immunoprecipitation of Panx1-flag was absent in HEK293T-P1KO cells transfected with *GFP* and *mPanx1-flag* cDNAs (right). **(D)** Immunoprecipitates from NMCs with anti-Piezo1 (right) or anti-Ki67 (left) antibodies. Immunoprecipitation was followed by immunoblots with the anti-Panx1 antibody. Three independent experiments. The arrow indicates Panx1.

We investigated potential interactions between Piezo1 and Panx1 by coimmunoprecipitation in HEK293T-P1KO cells expressing GFP-tagged mPiezo1 and flag-tagged mPanx1. Expression of epitope tagged proteins was confirmed by Western blotting using antibodies raised against the appropriate epitope tag. Piezo1 was detected as a monomeric ≈290-kD immunoreactive band, whereas Panx1 was detected as a double band of 42–44 and 44–47 kD (Fig. 7 A and Fig. S5 C). A similar band pattern has been observed on Western blotting for Panx1 in nine independent experiments, and may reflect differential S-nitrosylation and glycosylation states (Lohman et al., 2012). When an anti-flag antibody was used to immunoprecipitate flag-tagged Panx1, GFP-tagged mPiezo1 was found to be coimmunoprecipitated (Fig. 7 A). If Piezo1 is truly able to coassociate with Panx1, then it should also be possible to demonstrate the presence of Panx1 in samples immunoprecipitated using an anti-GFP antibody. To directly test this prediction, we performed a reciprocal immunoprecipitation protocol, using the same transfected HEK293T-P1KO cell lysates described above. When the anti-GFP antibody was used to immunoprecipitate GFP-Piezo1, flag-tagged mPanx1 was coimmunoprecipitated, consistent with an interaction (Fig. 7 B). Neither Piezo1 nor Panx1 immunoreactivity was detected in control experiments with a rabbit polyclonal antibody recognizing an unrelated epitope (anti-Ki67 antibody; Fig. 7 C, left). As additional controls, Panx1 immunoreactivity was not detected in anti-GFP immunoprecipitates from lysates of cells cotransfected with GFP and flag-tagged mPanx1 cDNAs (Fig. 7 C, right).

We further attempted to isolate Piezo1 immunocomplexes from native cholangiocytes, but this turned to be a challenge due to the low level of Piezo1 protein under natural expression levels. Therefore, we took advantage of the use of an immortalized cell line of murine cholangiocytes that retains characteristics similar to those of freshly isolated cholangiocytes (Ueno et al., 2003). When the Thermo Fisher Scientific anti-Piezo1 antibody was used to immunoprecipitate Piezo1, Panx1 was found to be present in Piezo1 immunocomplexes (Fig. 7 D).

Our findings therefore suggest that Piezo1 and Panx1 can be isolated in a stable complex by coimmunoprecipitation. To assess whether these complexes may localize in plasma membrane domains, we dually transfected Piezo1-GFP and Panx1-flag in HEK293T-P1KO cells. Subsequent analysis of the immunostained tags using confocal microscopy showed patterns of membrane colocalization (Fig. S5 D). Profile analysis across cotransfected cells showed dense fluorescence intensity at the cell periphery (Fig. S5 E). We confirmed the absence of detectable immunostaining in cells not expressing the cognate protein, demonstrating the specificity of both the primary and secondary antibodies (data not shown). The observed overlapping distribution is consistent with our prediction that these two proteins may interact in plasma membrane domains.

### Coexpression of mPiezo1, Panx1, and P2X4R reconstitutes a functional ATP-secreting protein complex

We cotransfected *mPiezo1-GFP*, *mPanx1-HA*, and *P2X4R-myc* cDNAs in HEK293T-P1KO cells to reconstitute functional complexes. Subsequent analysis of immunostained tags using confocal microscopy showed successful coexpression of the three proteins at the cell periphery (Fig. S6 A). Yoda1-induced Ca^2+^ responses in these cells were markedly augmented compared with HEK293T-P1KO cells expressing only mPiezo1-GFP or mPiezo1-GFP and mPanx1-HA (Fig. S6 B), suggesting stimulation of P2X4Rs by released ATP. Consistently, application of exogenous ATP caused a massive calcium signal (+218% on average), confirming the effective expression of P2X4Rs. On average, Yoda1 responses in cells expressing the three proteins were significantly increased by 119% and 45% compared with responses in cells expressing mPiezo1-GFP alone or mPiezo1-GFP and mPanx1-HA (Fig. S6 C). Moreover, apyrase reduced Yoda1 signals in cells coexpressing the three proteins, and produced overshooting Ca^2+^ responses on washout, indicative of ATP release and stimulation of P2X4Rs (Fig. S6 D).

## Discussion

**Figure 8.**
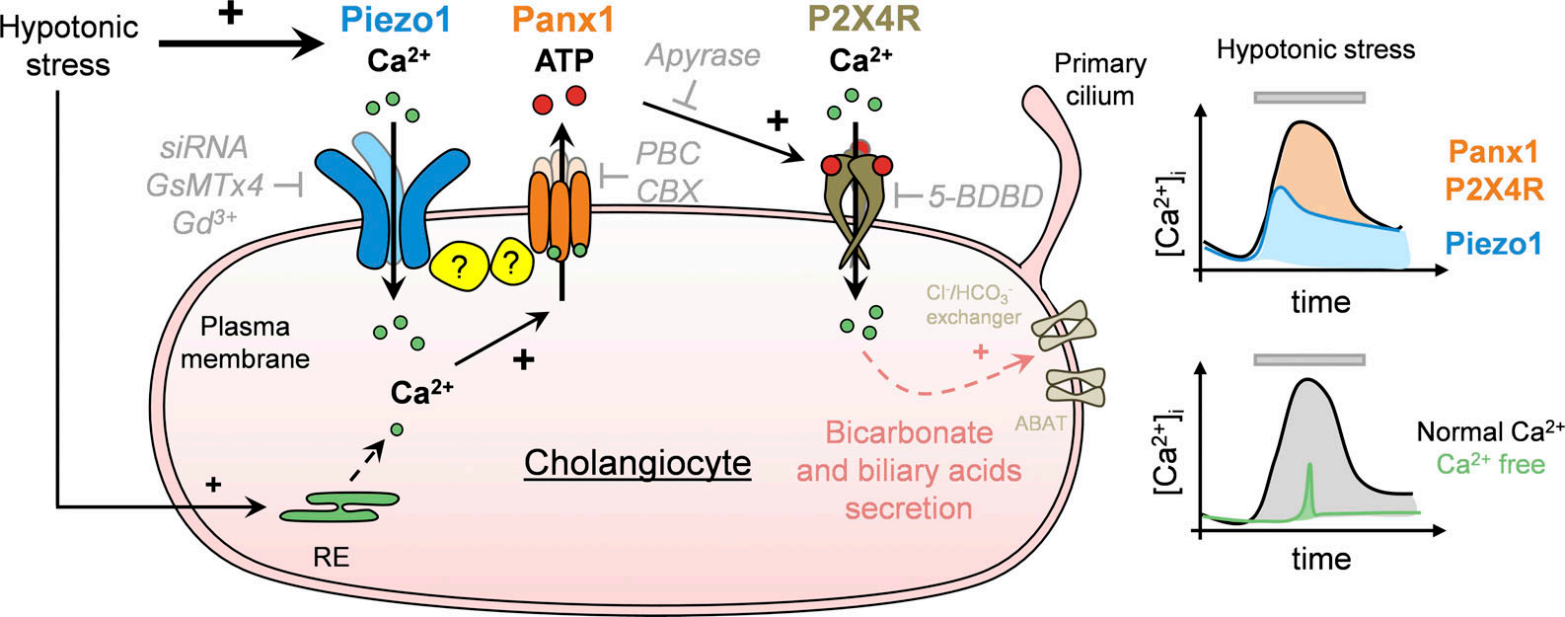
**Piezo1–Panx1 complex model for stretch-induced ATP release in cholangiocytes.** Hypotonic stress elevates intracellular calcium in cholangiocytes through a mechanism that depends on Ca^2+^ influx and secreted ATP. The cellular mechanism of regulated ATP release involves sequential activation of Piezo1 and Panx1. By mediating a rise in intracellular Ca^2+^, Piezo1 is responsible for translating membrane stretch into Panx1-mediated ATP secretion. Released ATP binds P2X4R in an autocrine/paracrine manner, which may influence transport processes and ductal bile secretion. RE, endoplasmic reticulum.

Using molecular, pharmacological, and functional approaches, the present study identifies a new pathway for osmosensation and ATP secretion in cholangiocytes. We show that Piezo1 serves as an osmosensor triggering [Ca^2+^]_i_ increase and subsequent activation of Panx1, which acts as ATP-conducting pathway. This chain of events leads to signal amplification through stimulation of autocrine/paracrine P2X4R. Collectively, these data provide a new working model for mechanosensitive secretion of ATP in bile ducts (Fig. 8).

### Hypotonic Ca^2+^ responses occur through cilium-independent pathways and P2X4R stimulation

Our data indicate that mouse cholangiocytes are sensitive to hypotonic but not hypertonic challenges. Hypotonic responses are based on membrane deformation following the swelling of the cell by water influx (Wang et al., 1996). At variance with previous work (Gradilone et al., 2007), we found that hypotonic Ca^2+^ responses do not require the primary cilium. This discrepancy may be related to the difference in methodological approaches or preparations. Nevertheless, there are many precedents for cilium-independent osmotic responses and ATP release in many cell types (Rodat-Despoix et al., 2013; Iomini et al., 2004), including cholangiocyte-derived cells (Woo et al., 2008). While our data suggest that hypotonic response occurs through a cilium-independent pathway, they do not discount a contribution of the primary cilium in the response to shear stress (Gradilone et al., 2007).

We found that hypotonic Ca^2+^ responses were essentially dependent on extracellular calcium. In a few instances, residual Ca^2+^ responses were observed in free [Ca^2+^]_o_, suggesting that P2YRs, linked to inositol triphosphate-mediated release of Ca^2+^ from intracellular endoplasmic reticulum stores, may contribute a minor component of the hypotonic Ca^2+^ response (Hirata et al., 2002; Dutta et al., 2008). Yet we found that propagation of Ca^2+^ waves from stimulated cholangiocytes to the neighboring cells prominently relies on the autocrine/paracrine action of ATP on P2X4R, the dominant subtype in rodent cholangiocytes (Doctor et al., 2005; Woo et al., 2010). P2R stimulation is known to increase transepithelial Cl^−^ secretion and to contribute to transport of water and HCO_3_^−^ in both rodent and human biliary epithelial models (Roman et al., 1999; Zsembery et al., 1998). Thus, stimulation of P2X4R may be an important regulatory step linking membrane-directed forces to coordinated cholangiocyte responses regulating bile formation and secretion.

### Piezo1 serves as an osmosensor in cholangiocytes

Piezo1 has been involved in mechanosensitive ATP release pathways in different cell systems (Cinar et al., 2015; Miyamoto et al., 2014; Wang et al., 2016; Diem et al., 2020). Here we showed that Piezo1 is an important determinant of mechanosensation in mouse cholangiocytes. Piezo1 is endowed with properties that make it a likely candidate for a cholangiocyte osmosensor (Coste et al., 2010; Ranade et al., 2015). Down-regulation of Piezo1 by siRNAs resulted in substantial inhibition of both hypotonic Ca^2+^ responses and ATP secretion. We obtained similar inhibitory effects with the spider toxin blocker GsMTx4, and gadolinium, although both might have off-targets. Additionally, activation of Piezo1 by Yoda1 induced long-lasting Ca^2+^ responses in cholangiocytes, which require ATP secretion and P2XR signaling. Collectively, these data indicate that Piezo1 contributes to hypotonic Ca^2+^ responses and ATP release in cholangiocytes.

A previous search for osmosensitive ion channels in cholangiocytes pointed to the involvement of TRPV4, located on the primary cilium (Gradilone et al., 2007). More recently, TRPV4 has also been shown to translate fluid flow into intracellular signaling and biliary secretion (Li et al., 2020). It is therefore plausible that both Piezo1 and TRPV4 serve as mechanosensors in cholangiocytes as neither Piezo1 (this study) nor TRPV4 (Gradilone et al., 2007) knock-down abolished hypotonic Ca^2+^ responses. Thus, the presence of different mechanosensors endows cholangiocytes with the aptitude to distinguish different mechanical stimuli, and eventually to signal them differentially.

### Piezo1–Panx1 complex couples force detection to ATP secretion

Our data indicate that Piezo1 is necessary but not sufficient for ATP secretion. Previous studies have proposed two nonexclusive mechanisms for ATP secretion in cholangiocytes, that is, exocytosis of ATP enriched vesicles and release through ATP-permeable CFTR channels (Fitz, 2007). Our investigation is suggestive of an additional mechanism involving Panx1-mediated ATP release. Supporting this idea, we found that pharmacological inhibition of Panx1 strongly reduced ATP secretion in response to both hypotonic stress and Yoda1 exposure. A broad set of experimental evidence indicates that Panx1 can mediate ATP release under physiological and pathological conditions in a variety of cell types (Dahl, 2015). Panx1 can form a transmembrane channel called pannexon, permitting ATP efflux from cells down its concentration gradient.

How might Piezo1 regulate the function of Panx1 channels? Prior studies have shown that an increase in cytoplasmic Ca^2+^ concentration can activate Panx1 (Locovei et al., 2006). A tantalizing hypothesis is therefore that calcium influx mediated by Piezo1 provides a triggering mechanism for Panx1 activation. This functional relationship may be enhanced by physical promiscuity. By a combination of immunocytochemistry and coimmunoprecipitation techniques, we were able to provide evidence that both proteins can colocalize and interact physically in recombinant systems and NMCs. Although these findings suggest an ability to interact, directly or through intervening proteins, we could not demonstrate whether the Piezo1–Panx1 complex exists in native cholangiocytes. Thus, whether Piezo1 physically interacts with Panx1 in native cholangiocytes will have to be resolved in further studies.

In conclusion, the present study was initiated to explore stretch-sensitive mechanisms and ATP release in cholangiocytes. Our data are consistent with the idea that both Piezo1 and Panx1 molecules are necessary for eliciting downstream effects of hypotonic stress. By mediating a rise in intracellular Ca^2+^, Piezo1 acts as a mechanosensor responsible for translating cell swelling into activation of Panx1, which is critical for ATP release. These findings provide new insight into the molecular mechanisms controlling ATP secretion and may facilitate the development of novel therapeutic strategies for liver diseases.

## Supplementary Material

Table S1 lists sequences of siRNA.

Table S2 provides sequences of RT-PCR primers.

Table S3 shows sequences of qPCR primers.

Table S4 shows detailed information depicting the numbers of independent experiments (*N*), the number of cells (*n*) analyzed in each individual experiment, and the corresponding averaged values.

Table S5 lists reagents and tools.
