# Supplementary material for: Piezo1–Pannexin1 complex couples force detection to ATP secretion in cholangiocytes
Source: J Gen Physiol. 2021 Oct 25;153(12):e202112871. doi: 10.1085/jgp.202112871 (PMC8548913; doi:10.1085/jgp.202112871)
Supplement: Table S1 — lists sequences of siRNA. [file JGP_202112871_TableS1.docx]

**Table S1. Sequences of siRNA**

|  | siPiezo1 |  | siScramble |
| --- | --- | --- | --- |
| Target  Sequences  (5’🡪 3’) | GAAAGAGAUGUCACCGCUA |  | UGGUUUACAUGUCGACUAA |
|  | GCAUCAACUUCCAUCGCCA |  | UGGUUUACAUGUUGUGUGA |
|  | AAAGACAGAUGAAGCGCAU |  | UGGUUUACAUGUUUUCUGA |
|  | GGCAGGAUGCAGUGAGCGA |  | UGGUUUACAUGUUUUCCUA |
