# Supplementary material for: Piezo1–Pannexin1 complex couples force detection to ATP secretion in cholangiocytes
Source: J Gen Physiol. 2021 Oct 25;153(12):e202112871. doi: 10.1085/jgp.202112871 (PMC8548913; doi:10.1085/jgp.202112871)
Supplement: Table S2 — provides sequences of RT-PCR primers. [file JGP_202112871_TableS2.docx]

**Table S2. Sequences of RT-PCR primers**

| Genes | Reference no. / Accession no. | Forward primer  (5’🡪 3’) | Reverse primer  (5’🡪 3’) | Size  (pb) |
| --- | --- | --- | --- | --- |
| CK-19 | NM_008471.3 | TTGCTCGGATTGAGGAGCTG | TTCATGCTGTGCTGGGACTG | 136 |
| CK-7 | NM_033073.3 | CTCTCGCTCCACTGCTTACC | CGACTGCAGCTCTGCTAACT | 679 |
| CFTR | NM_021050.2 | TGCGATCTGTGAGCAGAGTG | GCACATAACCCCCATCCACA | 570 |
| AE2 | NM_009207.3 | GGTGGATAGAGAGCGTGAGC | TGTCCTCCGAAGGGGATCAT | 598 |
| ASBT | NM_011388.3 | GGCTACAGCCTGGGTTTCTT | CACCAGGTTGAGATCCTCGG | 150 |
| Albumin | NM_009654.4 | CAGATGACAGGGCGGAACTT | AGGTGCTTTCTGGGTGTAGC | 488 |
| Piezo1 | NM_001357349.1 | GCTTTTGGGAAGCACTCTGC | CACAGAGCGGATCAGTGACA | 660 |
| Piezo1 | NM_001142864.4, NM_001357349.1 | TTCCCCAACAGCACCAACTT | GGCAGGTACAGCCACTTGAT | 596 |
| Piezo2 | NM_001039485.4 | CAGCCATGCCAAAGTCAATGG | CCACTTGTTCCTGAGGTCTGA | 277 |
| TRPV2 | NM_011706.2 | ATGCTTAGAACTAAGGTGGAGG | AGAGTCGGTCACGGTCAAAC | 498 |
| TRPV4 | NM_022017.3 | CCTGCTCTTCATGATCGGCT | AGACCAGTTCACCTCGTCCA | 496 |
| PKD1 | NM_013630.2 | GCAGCTTCAGTGTGGTCTCT | GAAGATGGCAGCACTCTGGT | 539 |
| PKD2 | NM_008861.3 | TGGAAGTTCACCGAAGGCTC | AGCCCCTCTTCATTTGACAT | 767 |
| P2rX1 | NM_008771.3 | GACAAACCGTCGTCACCTCT | CCCATGTCCTCCGCATACTT | 222 |
| P2rX2 | NM_001310701.1 | TGGACAGGCAGGGAAATTCA | CAAGACAAGGGCAAGGGTCA | 208 |
| P2rX4 mouse | NM_011026.3 | TGTGGCTGTGACCAACACTT | GTCAAACTTGCCAGCCTTTCC | 784 |
| P2rX4 human | NM_001256796.2 | GTCAGCTCCGTTACGACCAA | CGTCTTGACAGACCCGTTGA | 294 |
| P2rX5 | NM_033321.3 | CCCACTGCAACCCACACTAT | ATTCTCCTGGAGGCCAGACC | 509 |
| P2rX7 | NM_001038839.2 | TTATGGCACCGTCAAGTGGG | GCCTGGGATACTCAGGACAC | 299 |
| Panx1 | NM_019482.2 | AGATCTCCATCGGTACCCAGA | GTGGGAGGTTTCCAGACTCG | 138 |
