# Supplementary material for: Piezo1–Pannexin1 complex couples force detection to ATP secretion in cholangiocytes
Source: J Gen Physiol. 2021 Oct 25;153(12):e202112871. doi: 10.1085/jgp.202112871 (PMC8548913; doi:10.1085/jgp.202112871)
Supplement: Table S3 — shows sequences of qPCR primers. [file JGP_202112871_TableS3.docx]

**Table S3. Sequences of qPCR primers**

| Transcript | Reference no. / accession no. | Forward primer (5’🡪 3’) | Reverse primer (5’🡪 3’) | Size  (pb) |
| --- | --- | --- | --- | --- |
| Piezo1 | NM_001357349.1 | ATCGCCATCATCTGGTTCCC | CTAGCTTGAGGGTGACGGTG | 100 |
| CK-19 | NM_008471.3 | AAGACCATCGAGGACTTGCG | GCGTGTTCTGTCTCAAACTTGG | 128 |
| GAPDH | XM_017321385.1 | GCAAATTCAACGGCACA | CACCAGTAGACTCCACGAC | 141 |
| β-actin | NM_007393.5 | GCCAACCGTGAAAAGATGAC | GGCGTGAGGGAGAGCATAG | 183 |
