# Supplementary material for: Piezo1–Pannexin1 complex couples force detection to ATP secretion in cholangiocytes
Source: J Gen Physiol. 2021 Oct 25;153(12):e202112871. doi: 10.1085/jgp.202112871 (PMC8548913; doi:10.1085/jgp.202112871)
Supplement: Table S4 — shows detailed information depicting the numbers of independent experiments (N), the number of cells (n) analyzed in each individual experiment, and the corresponding averaged values. [file JGP_202112871_TableS4.docx]

**Supplemental Table 4.**

Detailed information depicting the numbers of independent experiments (*N*), the number of cells (*n*) analyzed in each individual experiment, and the corresponding averaged values. Units are not reported and can be found in the figures.

| Figure panels | | Independent tests (*N*) | Conditions | Number of cells (*n*) in each *N* and corresponding mean value or % of responsive cells |
| --- | --- | --- | --- | --- |
| 1 | E | 5 | 2.5 mM Ca^2+^ | *n* = 59, 77, 71, 137, 135  % = 98.3, 85.71, 78.87, 59.85, 25.18 |
|  |  |  | 0 Ca^2+^ | *n* total = 24, 41, 40, 52, 80  % = 25.00, 73.17, 30.00, 15.38, 10.00 |
|  | F | 5 | 2.5 mM Ca^2+^ | *n* = 58, 66, 56, 82, 34  mean = 1.02, 0.97, 0.61, 0.47, 0.42 |
|  |  |  | 0 Ca^2+^ | *n* = 6, 30, 12, 8, 8  mean = 0.32, 0.55, 0.51, 0.27, 0.35 |
|  | G | 5 | 2.5 mM Ca^2+^ | *n* as in F  mean = 4.07, 3.81, 2.74, 2.10, 1.47 |
|  |  |  | 0 Ca^2+^ | *n* as in F  mean = 1.39, 1.22, 1.57, 0.62, 1.14 |
|  | H | 4 | 2.5 mM Ca^2+^ | *n* = 66, 56, 82, 34  mean = 1.45, 0.47, 0.24, 0.88, 0.90 |
|  |  |  | 0 Ca^2+^ | *n* = 30, 12, 8, 8  mean = 0.29, 0.34, 0.04, 0.21 |
|  | I | 4 | 2.5 mM Ca^2+^ | *n* as in H  mean = 2.64, 0.99, 0.88, 1.85 |
|  |  |  | 0 Ca^2+^ | *n* as in H  mean = 0.61, 0.84, 0.16, 0.73 |
| 2 | B | 3 | Ctrl | *n* = 197, 139, 147  % = 67.01, 94.96, 61.90 |
|  |  |  | Apyrase | *n* = 52, 287, 124  % = 46.15, 60.63, 51.61 |
|  | C | 3 | Ctrl | *n* = 92, 131, 132  mean = 0.67, 0.87, 0.56 |
|  |  |  | Apyrase | *n* = 64, 174, 24  mean = 0.47, 0.54, 0.28 |
|  | D | 3 | Ctrl | *n* as in C  mean = 2.68, 3.37, 2.54 |
|  |  |  | Apyrase | *n* as in C  mean = 1.59, 2.08, 1.19 |
|  | F | 3 | Ctrl | *n* = 81, 66, 61  mean = 0.47, 0.41, 0.63 |
|  |  | 3 | Apyrase | *n* = 119, 79, 114  mean = 0.02, 0.01, 0.06 |
|  | I | 3 | Ctrl | *n* = 173, 164, 191  % = 87.86, 59.15, 84.29 |
|  |  | 4 | A-804598 | *n* = 186, 130, 96, 106  % = 75.27, 69.23, 75.00, 85.84 |
|  |  | 4 | A-740003 | *n* = 216, 172, 85, 85  % = 79.17, 60.47, 83.53, 77.65 |
|  | J | 3 | Ctrl | *n* = 152, 97, 161  mean = 0.90, 1.07, 1.00 |
|  |  | 4 | A-804598 | *n* = 140, 90, 72, 91  mean = 1.13, 0.94, 1.12, 1.30 |
|  |  | 4 | A-740003 | *n* = 171, 104, 71, 66  mean = 1.17, 0.93, 1.09, 0.66 |
|  | K | 3 | Ctrl | *n* as in J  mean = 3.42, 2.98, 3.76 |
|  |  | 4 | A-804598 | *n* as in J  mean = 3.68, 3.54, 2.71, 4.96 |
|  |  | 4 | A-740003 | *n* as in J  mean = 4.20, 3.49, 3.53, 2.13 |
|  | L | 3 | Ctrl | *n* as in I  mean = 2.15, 1.91, 2.86 |
|  |  | 4 | A-804598 | *n* as in I  mean = 1.97, 2.26, 1.95, 3.28 |
|  |  | 4 | A-740003 | *n* as in I  mean = 1.81, 1.97, 2.16, 2.27 |
|  | N | 3 | Ctrl | *n* = 86, 105, 74  % = 97.67, 75.24, 52.70 |
|  |  | 3 | 5-BDBD | *n* = 98, 115, 69  % = 88.78, 83.47, 56.52 |
|  | O | 3 | Ctrl | *n* = 83, 79, 39  mean = 1.12, 0.74, 0.81 |
|  |  |  | 5-BDBD | *n* = 87, 96, 39  mean = 0.51, 0.46, 0.45 |
|  | P | 3 | Ctrl | *n* as in O  mean = 3.57, 1.39, 1.09 |
|  |  |  | 5-BDBD | *n* as in O  mean = 1.08, 1.43, 1.23 |
|  | Q | 3 | Ctrl | *n* as in N  mean = 1.50, 1.22, 1.19 |
|  |  | 3 | 5-BDBD | *n* as in N  mean = 0.67, 0.74, 0.89 |
| 3 | B | 3 | Ctrl | *n* = 245, 125, 163  % = 69.79, 38.40, 26.38 |
|  |  |  | Gd^3+^ | *n* = 246, 156, 128  % = 18.29, 14.10, 12.50 |
|  | C | 3 | Ctrl | *n* = 171, 48, 43  mean = 0.61, 0.47, 0.48 |
|  |  |  | Gd^3+^ | *n* = 45, 22, 16  mean = 0.47, 0.38, 0.47 |
|  | D | 3 | Ctrl | *n* as in C  mean = 3.05, 1.81, 1.55 |
|  |  |  | Gd^3+^ | *n* as in C  mean = 3, 2.08, 1.27, 1.50 |
|  | H | 2 | 10 µM | *n* = 136, 67  mean = 0.33, 0.49 |
|  |  |  | 50 µM | *n* = 130, 148  mean = 1.42, 0.91 |
|  |  |  | 100 µM | *n* = 139, 116  mean = 1.39, 0.97 |
|  | K | 3 | GFP | *n* = 33, 99, 67  mean = 0.07, 0.09, 0.07 |
|  |  | 2 | Piezo1 – 50 µM | *n* = 52, 46  mean = 0.58, 0.41 |
|  |  | 1 | Piezo1 – 100 µM | *n* = 59 ; mean = 1.01 |
| 4 | C |  | siCtrl | *n* = 239, 104, 69  % = 92.88, 95.19, 86.95 |
|  |  |  | siPiezo1 | *n* = 210, 108, 66  % = 90.00, 50.00, 80.30 |
|  | D | 3 | siCtrl | *n* = 222, 99, 60  mean = 1.15, 0.58, 0.73 |
|  |  |  | siPiezo1 | *n* = 189, 54, 53  mean = 0.74, 0.46, 0.48 |
|  | F | 3 | siCtrl | *n* = 171, 136, 165  % = 92.39, 92.64, 84.84 |
|  |  |  | siPiezo1 | *n* total = 193, 198, 222  % = 81.34, 78.39, 83.33 |
|  | G | 3 | siCtrl | *n* = 157, 166, 124  mean = 0.61, 0.70, 0.61 |
|  |  |  | siPiezo1 | *n* = 157, 170, 185  mean = 0.57, 0.55, 0.48 |
|  | H |  | siCtrl | *n* as in G  mean = 3.67, 4.15, 5.50 |
|  |  |  | siPiezo1 | *n* as in G  mean = 3.42, 3.01, 4.23 |
|  | J | 3 | GFP | *n* = 29, 52, 41  mean = 0.12, 0.16, 0.07 |
|  |  |  | Piezo1 | *n* = 45, 18, 36  mean = 0.31, 0.43, 0.57 |
|  | K | 3 | GFP | *n* as in J  mean = 0.19, 0.20, 0.21 |
|  |  |  | Piezo1 | *n* as in J  mean = 0.16, 0.35, 0.25 |
| 5 | E | 2 | Yoda1 | *n* = 246, 227  % = 80.49, 97.36 |
|  |  |  | Yoda1+apyrase | *n* = 233, 215  % = 63.24, 96.28 |
|  | F | 2 | Yoda1 | *n* = 198, 221  mean = 0.63, 1.21 |
|  |  |  | Yoda1+apyrase | *n* = 159, 207  mean = 0.42, 0. 94 |
|  | H | 2 | 10 min | *n* = 108, 95  mean = 0.49, 0.36 |
|  |  |  | 13 min | *n* = 59, 29  mean = 0.19, 0.14 |
| 6 | C | 3 | Ctrl | *n* = 166, 129, 95  % = 93.37, 97.67, 89.47 |
|  |  |  | PBC | *n* = 251, 155, 143  % = 90.44, 92.25, 60.84 |
|  | D | 3 | Ctrl | *n* = 157, 127, 85  mean = 0.76, 0.73, 0.45 |
|  |  |  | PBC | *n* = 227, 143, 87  mean = 0.51, 0.59, 0.40 |
|  | E | 3 | Ctrl | *n* as in D  mean = 4.12, 4.50, 3.12 |
|  |  |  | PBC | *n* as in D  mean = 2.91, 3.44, 2.77 |
|  | F | 3 | Ctrl | *n* = 299, 292, 176  % = 85.28, 91.09, 90.91 |
|  |  |  | CBX | *n* = 249, 209, 185  % = 74.69, 42.10, 28.10 |
|  | H | 3 | Yoda1 | *n* = 278, 237, 158  % = 98.50, 92.40, 76.58 |
|  |  |  | Yoda1+PBC | *n* = 305, 289, 170  % = 95.73, 90.65, 61.76 |
|  | I | 3 | Yoda1 | *n* = 121, 219, 274  mean = 0.63, 0.59, 1.05 |
|  |  |  | Yoda1+PBC | *n* = 105, 262, 292  mean = 0.53, 0.62, 0.72 |
|  | K | 2 | Yoda1 | *n* = 131, 207  mean = 0.64, 0.74 |
|  |  |  | Yoda1+PBC | *n* = 68, 115  mean = 0.40, 0.94 |

| S1 | B | 2 | DIV 2 | *n* = 456, 632  % of CK19^+^ cells = 53.42, 50.15 |
| --- | --- | --- | --- | --- |
|  |  | 4 | DIV 5 | *n* = 631, 102, 318, 511  % of CK19^+^ cells = 88.70, 67.51, 72.55, 86.10 |
|  |  | 3 | DIV 8 | *n* = 602, 788, 951  % of CK19^+^ cells = 86.53, 69.61, 87.33 |
|  |  | 3 | DIV 12 | *n* = 73, 81, 577  % of CK19^+^ cells = 75.06, 15.15, 51.35 |
|  | E | 2 | Krebs | *n* = 68, 26  mean = 1.14, 1.01 |
|  |  |  | HYPER | mean = 1.03, 0.99 |
|  |  |  | ATP | mean = 1.93, 1.74 |
| S2 | B | 2 | DIV 2 | *n* = 297, 342  % of CK19^+^ ciliated cells = 10.26, 15.15 |
|  |  | 4 | DIV 5 | *n* = 546, 238, 73, 486  % of CK19^+^ ciliated cells = 28.28, 29.32, 22.97, 29.96 |
|  |  | 3 | DIV 8 | *n* = 534, 606, 731  % of CK19^+^ ciliated cells = 21.80, 19.72, 19.89 |
|  |  | 3 | DIV 12 | *n* = 113, 217, 39  % of CK19^+^ ciliated cells = 21.23, 20, 10.23 |
|  | D | 3 | Ctrl | *n* = 637, 319, 446  % of CK19^+^ cells = 80.59, 76.07,86.06 |
|  |  |  | Chloral hydrate | *n* = 519, 430, 209  % of CK19^+^ cells = 92.17, 78.53, 93.49 |
|  | E | 3 | Ctrl | *n* = 809, 329, 184  % of CK19^+^ ciliated cells = 26.37, 17.02, 29.89 |
|  |  |  | Chloral hydrate | *n* total = 480, 257, 431  % of CK19^+^ ciliated cells = 12.70, 6.22, 9.28 |
|  | G | 3 | Ctrl | *n* = 152, 108, 195  % = 51.32, 93.52, 38.97 |
|  |  |  | Chloral hydrate | *n* total = 122, 92, 195  % = 63.93, 69.57, 40.00 |
|  | H | 3 | Ctrl | *n* = 78, 101, 76  mean = 0.51, 0.60, 0.41 |
|  |  |  | Chloral hydrate | *n* = 71, 64, 78  mean = 0.43, 0.57, 0.49 |
|  | I | 3 | Ctrl | *n* as in G  mean = 1.08, 1.40, 0.89 |
|  |  |  | Chloral hydrate | *n* as in G  mean = 0.76, 0.74, 0.60 |
|  | K | 2 | Ctrl | *n* = 52, 69  mean = 0.49, 0.58 |
|  |  |  | Suramin 20 µM | *n* = 35, 43  mean = 0.60, 0.57 |
|  |  |  | Suramin 200 µM | *n* = 16, 22  mean = 0.34, 0.38 |
| S4 | B | 2 | Yoda1 | *n* = 131, 207  mean = 0.64, 0.74 |
|  |  | 3 | Yoda1+apyrase | *n* = 109, 136, 53  mean = 0.41, 1.38, 0.75 |
| S6 | C | 2 | Piezo1 | *n* = 21, 15  mean = 0.36, 0.39 |
|  |  |  | Piezo1 + Panx1 | *n* = 33, 19  mean = 0.58, 0.54 |
|  |  |  | Piezo1 + Panx1 + P2X4R | *n* = 41, 34  mean = 0.74, 0.89 |
