# Supplementary material for: Piezo1–Pannexin1 complex couples force detection to ATP secretion in cholangiocytes
Source: J Gen Physiol. 2021 Oct 25;153(12):e202112871. doi: 10.1085/jgp.202112871 (PMC8548913; doi:10.1085/jgp.202112871)
Supplement: Table S5 — lists reagents and tools. [file JGP_202112871_TableS5.docx]

**Table S5. Reagents and tools**

| Reagent/resource | Reference or source | Identifier or catalog number |
| --- | --- | --- |
| **Experimental models** |  |  |
| C57Bl6 mice | Janvier Labs and inbred colonies | https://www.janvier-labs.com/fiche_produit/souris-c57bl-6jrj/ |
| HEK293t cells | American Type Culture Collection | Cat# CRL-3216 |
| HEK.Piezo1KO cells | A. Patapoutian, Scripps Research Institute | Lukacs et al., 2015 |
| NMC cells | Shannon Glaser, Texas A&M University System Health Science Center | N/A |
| **Recombinant DNA** |  |  |
| Mouse Piezo1iresGFP | This paper | N/A |
| Mouse Piezo1-GFP | A. Patapoutian | - |
| pCMV3-N-GFPspark | Interchim | Cat# CV027 |
| Mouse Panx1-flag | Interchim | Cat# MG52303-CF |
| **Antibodies** *IF: immunofluorescence; IP: immunoprecipitation; WB: Western blot* |  |  |
| Rabbit monoclonal anti-CK-19 IF (1:200) | Abcam | Cat# ab52625 |
| Goat polyclonal anti-albumin  IF (1:200) | Euromedex | Cat# A90-234A |
| Mouse monoclonal anti-acetylated tubulin  IF (1:200) | Sigma-Aldrich | Cat# T6793 |
| Rabbit polyclonal anti-piezo1 | Thermo Fisher Scientific | Cat# PA5-77617 |
| IF (1:100) |  |  |
| IP (7 µg/ml of beads) |  |  |
| WB (not specific) |  |  |
| Rabbit polyclonal anti-piezo1 | Proteintech | Cat# 15939-1-AP |
| IF (1:50 human isoform) |  |  |
| WB (not specific) |  |  |
| Rabbit polyclonal anti-piezo1 | Alomone | Cat# APC-087 |
| IF and WB (not specific) |  |  |
| Rabbit polyclonal anti-piezo1 | Origen | Cat# TA309651 |
| IF and WB (not specific) |  |  |
| Rabbit polyclonal anti-piezo1 | Novus | Cat# NBP1-78537 |
| IF and WB (not specific) |  |  |
| Rabbit polyclonal anti-piezo1 | Abgent | Cat# AG1563 |
| IF and WB (not specific) |  |  |
| Rabbit polyclonal anti-panx1 | Alomone | Cat# ACC-234 |
| IF (1:300) |  |  |
| WB (1:400) |  |  |
| Rabbit polyclonal anti-Ki67  IP (7 or 10 µg/ml of beads) | Abcam | Cat# ab15580 |
| rabbit polyclonal anti-GFP | Abcam | Cat# ab6556 |
| IF (1:200) |  |  |
| IP (5 µg/ml of beads) |  |  |
| WB (1:1,000) |  |  |
| Mouse polyclonal anti-GFP | AbD Serotech | Cat# OCT1820 |
| IF (1:200) |  |  |
| WB (1:1,000) |  |  |
| Rabbit polyclonal anti-flag | Abcam | Cat# ab1162 |
| IF (1:200) |  |  |
| IP (10 µg/ml of beads) |  |  |
| WB (1:1,000) |  |  |
| Mouse monoclonal anti-flag | Sigma-Aldrich | Cat# F1804 |
| IF (1:100) |  |  |
| WB (1:1,000) |  |  |
| TRITC-coupled polyclonal donkey anti-rabbit | Jackson ImmunoResearch | Cat# 711-025-152 |
| IF (1:1,000) |  |  |
| Alexa 488–coupled polyclonal donkey anti-goat | Invitrogen | Cat# A11055 |
| IF (1:1,000) |  |  |
| Alexa 488–coupled polyclonal donkey anti-mouse | Invitrogen | Cat# A21202 |
| IF (1:1,000) |  |  |
| Alexa 647–coupled polyclonal donkey anti-mouse | Invitrogen | Cat# A-31571 |
| IF (1:1,000) |  |  |
| HRP-coupled goat anti-mouse | BioRad | Cat# 170-6516 |
| WB (1:1,000) |  |  |
| HRP-coupled goat anti-rabbit | BioRad | Cat# 170-6515 |
| WB (1:1,000) |  |  |
| **Oligonucleotides and other sequence-based reagents** |  |  |
| piezo1-siRNA (SMART pool ON-TARGETplus) | Dharmacon | Cat# L-061455-00 |
| Scrambled siRNA (ON-TARGETplus Non-targeting pool) | Dharmacon | Cat# D-001810-10 |
| siGLO Red Transfection Indicator | Dharmacon | Cat# D-001630-02 |
| Custom DNA Oligo | Eurofins Genomics | This paper |
| **Chemicals, enzymes and other reagents** |  |  |
| Fura-2-AM | Molecular Probes | Cat# F1221 |
| Lipofectamine 2000 | Invitrogen | Cat# 11668 |
| Lipofectamine RNAimax | Invitrogen | Cat# 13778 |
| 5-BDBD | Sigma-Aldrich | Cat# SML0450 |
| Probenecid | Sigma-Aldrich | Cat# P8761 |
| A-804598 | Sigma-Aldrich | Cat# SML0617 |
| A-740003 | Sigma-Aldrich | Cat# A0862 |
| ATP | Sigma-Aldrich | Cat# A9187 |
| Chloral hydrate | Sigma-Aldrich | Cat# C8383 |
| Gadolinium | Sigma-Aldrich | Cat# G7532 |
| Apyrase | Sigma-Aldrich | Cat# A7646 |
| Suramin | Sigma-Aldrich | Cat# S2671 |
| CBX | Sigma-Aldrich | Cat# C4790 |
| Yoda1 | Sigma-Aldrich | Cat# SML1558 |
| GsMTx4 | Smartox Biotechnology | Cat# 08GSM001 |
| Cold fish gelatin | Sigma-Aldrich | Cat# G7765 |
| Phalloidin-FITC | Sigma-Aldrich | Cat# P5282 |
| Trizol Reagent | Life Technologies | Cat# 15596026 |
| DNase I | Invitrogen | Cat# 18068 |
| Taq DNA polymerase | Invitrogen | Cat# 10342020 |
| Protein A magnetic beads | BioRad | Cat# 161-0411 |
| N-ethylmaleimide | Sigma-Aldrich | Cat# E3876 |
| Protease inhibitors with EDTA | Roche | Cat# 04 693 116 001 |
| Protease inhibitors without EDTA | Roche | Cat# 11836170001 |
| 3–8% precasted polyacrylamide gel | Thermo Fisher Scientific | Cat# EA03785BOX |
| 4–12% precasted polyacrylamide gel | Thermo Fisher Scientific | Cat# WG1401BOX |
| Nitrocellulose membrane | GE Healthcare Life Science | Cat# 10600002 |
| Chemiluminescence substrate POD | Roche | Cat# 11500694001 |
| Collagenase D | Sigma-Aldrich | Cat# COLLD-RO |
| Pronase | Sigma-Aldrich | Cat# PRON-RO |
| DNase | Sigma-Aldrich | Cat# DN25 |
| Hyaluronidase | Sigma-Aldrich | Cat# H3506 |
| Poly-D-lysin precoated coverslips | Corning | Cat# 354086 |
| Rat tail collagen type I | Corning | Cat# 354236 |
| Gentamicin | Gibco | Cat# 15750-037 |
| Vetflurane (isoflurane 1,000 mg/g) | Virbac | Cat# GTIN 03597132002653 |
| **Software** |  |  |
| ImageJ | - | https://imagej.nih.gov/ij/ |
| Xcellence RT | Olympus | https://xcellence-rt.software.informer.com/download/ |
| ZEN | Zeiss | https://www.zeiss.fr/microscopie/produits/microscope-software/zen-lite.html |
| GraphPad; prism 7.00 | GraphPad Software Inc. | https://www.graphpad.com/ |
| MASCOT software | Matrix Science | https://www.matrixscience.com/ |
| **Other** |  |  |
| SuperScriptIII First-Strand Synthesis System kit | Invitrogen | Cat# 18080-051 |
| NucleoSpin RNA kit | Macherey-Nagel | Cat# 740955.50 |
| Kapa Sybr Fast qPCR kit | Kapa Biosystems | Cat# KK4601 |
| Luciferinluciferase detection assay | Sigma-Aldrich | Cat# FLAAM |
| Dynabeads coimmunoprecipitation kit | Invitrogen | Cat# 14321D |
